# Modified Xiaoyaosan Regulates the Neuro-Immune Microenvironment in Chronic Stress: A Preclinical Meta-Analysis and NF-kappaB Pathway Mechanism Exploration

**DOI:** 10.3390/biomedicines14102270

**Published:** 2026-10-08

**Authors:** Yingying Liang, Kangli Dai, Kaixin Liu, Yuhan Yang, Yan Li, Yongmei Xie

**Affiliations:** 1 School of Health Preservation and Rehabilitation, Guizhou University of Traditional Chinese Medicine, Guiyang 550025, China; 2 Yuechi County Traditional Chinese Medicine Hospital, Guang’an 638300, China; 3 State Key Laboratory of Biotherapy and Cancer Center, Collaborative Innovation Center of Biotherapy, West China Hospital, Sichuan University, Chengdu 610041, China

**Keywords:** modified Xiaoyaosan, chronic unpredictable mild stress, hyperprolactinemia, NF-κB pathway, neuro-immune microenvironment, dopaminergic neurons

## Abstract

**Background**: Chronic unpredictable mild stress (CUMS) triggers systemic pathologies, notably depression and endocrine disorders such as hyperprolactinemia (HPRL). Xiaoyaosan (XYS), a classic traditional Chinese medicine formulation, has been proven effective in treating these stress-induced diseases. However, the precise molecular mechanisms linking its systemic endocrine regulation remain unclear. This study aims to evaluate the multi-target mechanisms of XYS through a combined meta-analytical and experimental approach. **Methods**: First, a meta-analysis of 25 preclinical studies comprising 569 animals evaluated the efficacy and underlying mechanisms of XYS in CUMS-induced models. Concurrently, an in vivo HPRL rat model was innovatively established using CUMS combined with an intraperitoneal injection of metoclopramide to investigate the pharmacological mechanisms of XYS. Hypothalamic tissues and serum were analyzed using ELISA, Western blot, Real-Time PCR, and immunofluorescence. **Results**: The meta-analysis demonstrated that XYS significantly ameliorates CUMS-induced weight loss, behavioral despair and anhedonia, with therapeutic mechanisms heavily linked to regulating neuroinflammation and the hypothalamic–pituitary–adrenal axis. Corroborating these systemic benefits, our in vivo experiments mechanistically revealed that XYS attenuated the expression of glial response markers, specifically microglia (IBA-1) and astrocytes (GFAP). XYS also downregulated the expression of iNOS, NADPH-Oxidase 2, and Dopamine D2 receptor, while upregulating Cryab closely associated with the NF-κB inflammatory cascade. This modulation coincided with suppressed secretion of pro-inflammatory cytokines, restoring tyrosine hydroxylase (TH) positive expression levels in the hypothalamic dopaminergic microenvironment. **Conclusions**: XYS functioned as a multi-target systemic regulator by modulating factors associated with the NF-κB/Cryab inflammatory cascade. This synergistic buffering of the neuro-immune microenvironment assisted in maintaining TH expression and restoring systemic equilibrium, providing strong preclinical evidence for XYS as a comprehensive preventive strategy against the onset of stress-induced mood and endocrine disorders.

## 1. Introduction

Chronic stress is an increasingly recognized pathological factor that triggers a cascade of systemic issues, particularly chronic wasting diseases, mood disorders, and endocrine dysfunctions [1]. To study these stress-induced pathologies, chronic unpredictable mild stress (CUMS) is widely recognized worldwide as a standard method for establishing animal models of depression [2]. Negative chronic stress, such as depression, is a serious and increasingly common mental illness characterized by persistent sadness and a loss of interest, affecting approximately 5% of adults worldwide [3]. Furthermore, negative chronic stress can impact the hypothalamus and amygdala, leading to a decrease in dopamine (DA) secretion, which is a primary trigger for endocrine disorders such as hyperprolactinemia (HPRL) [4,5,6]. HPRL is a prevalent gynecological endocrine disorder that leads to abnormally high prolactin (PRL) levels, severe reproductive dysfunction, and is closely associated with chronic stress [7,8]. Despite the severe burden these stress-induced behavioral and endocrine disorders place on global health, current pharmacological treatments often present challenges, including low oral bioavailability, significant dose-limiting toxicities, and high relapse rates.

In response to these challenges, Traditional Chinese Medicine (TCM) has garnered significant attention for its safety, cost-effectiveness, and multi-target therapeutic potential. Xiaoyaosan (XYS) is a classic TCM formulation, originating in the Song Dynasty, that is commonly used clinically to treat depression by harmonizing the liver and the spleen. Modified Xiaoyaosan (MXYS), derived from this classic prescription, has proven effective in alleviating immune-related diseases induced by CUMS and has demonstrated remarkable efficacy in mitigating HPRL in several studies [9,10,11]. MXYS retains the entire foundational botanical matrix of classic XYS, ensuring that its primary bioactive constituents modulate the inflammatory response, neuroplasticity, and the hypothalamic–pituitary–adrenal (HPA) axis, which remains conserved and pharmacologically dominant [12,13]. Because this core chemical profile is preserved, the well-documented immunomodulatory mechanisms of classic XYS serve as a highly relevant mechanistic baseline for MXYS.

To bridge this gap and provide strong preclinical evidence for clinical application, this study utilizes a comprehensive, dual-evidence design. First, a systematic evaluation and meta-analysis was conducted to assess the macroscopic efficacy and multi-pathway mechanisms of XYS in treating standard CUMS-induced depressive models. Concurrently, to simulate a complex clinical pathogenesis where stress intersects with endocrine dysfunction, we utilized an in vivo “dual-hit” model, CUMS combined with metoclopramide-induced HPRL, to investigate the pharmacological mechanisms of MXYS. By pairing the broad neuro-immune evidence of the meta-analysis with the targeted experimental exploration of the NF-κB inflammatory pathway, the present study aimed to elucidate the preventive potential of MXYS, determining how early modulation of the systemic immune network buffered the dopaminergic microenvironment against the development of overlapping psychological and pharmacological stressors.

## 2. Materials and Methods

### 2.1. Phase 1: Meta-Analysis

#### 2.1.1. Search Strategy & Literature Selection

A comprehensive literature search was conducted up to June 2026 across multiple databases, including the Cochrane Library, PubMed, Web of Science, EMbase, CNKI, VMIS, Wan-Fang, and CBM. The details for the search strategy and literature selection were shown in the Supplementary Methods. The inclusion criteria for the preclinical studies were as follows:

Population (P): Animal models of depression established by CUMS.

Intervention (I): Continuous treatment with XYS.

Control (C): Model groups that did not receive any treatment.

Outcome (O): Primary outcomes included body weight, food intake, sucrose preference test (SPT), open field test (OFT), forced swimming test (FST), and novelty suppressed feeding (NSF); secondary outcomes focused on the protective mechanism of XYS on depression.

#### 2.1.2. Data Extraction & Quality Evaluation

Data extracted from the included studies consisted of the first author’s name, publication year, animal species/sex/number, anesthetic used, modeling and administration details (time, dose, route), and outcome indices. Two researchers independently evaluated the methodological quality using the Collaborative Approach to Meta-Analysis and Review of Animal Data from Experimental Studies (CAMARADES) 10-point scoring scale, modified to include the avoidance of neuroprotective or neurotoxic anesthetics. More details for data extraction and methodological quality evaluation were shown in the Supplementary Methods.

#### 2.1.3. Statistical Analysis for Meta-Aalysis

Stata 15 software was utilized for meta-analysis. The standardized mean difference (SMD) was calculated with a 95% confidence interval (CI). Heterogeneity was evaluated using the I-squared test; a fixed-effect model was used if there was no significant heterogeneity, whereas a random-effect model was adopted if heterogeneity was present. Sensitivity and subgroup analyses were also performed to assess data robustness and publication bias.

### 2.2. Phase 2: Experimental Model

#### 2.2.1. Animals

We obtained 120 female Sprague-Dawley rats (200 ± 20 g) from the Fourth Military Medical University of the Chinese People’s Liberation Army [animal license No. SCXK (Chongqing) 2017–0011]. The rats were assigned at random into six groups of 20 rats each: control group, model group, bromocriptine group, high-dose XYS group, medium-dose XYS group, and low-dose XYS group. Research has indicated that psychological stress plays a critical role in the etiology of numerous endocrine illnesses. Therefore, to mimic clinical pathogenesis, a CUMS paradigm was integrated into the established pharmacological model. The CUMS procedure utilized in this study followed a highly standardized and globally recognized protocol designed to induce robust depression-like behavioral and neuro-immune deficits.

All groups except for the control group were subjected to CUMS for 21 days concurrently with metoclopramide administration. The specific stressors and their precise continuous durations were defined as follows: (1) complete food and water deprivation for 24 h; (2) water deprivation with free access to food for 24 h; (3) food deprivation with free access to water for 24 h; (4) forced swimming in ice water (4–8 °C) for 5 min; (5) damp bedding (200 mL of water poured into the cage sawdust) for 24 h; (6) physical restraint in a well-ventilated cylindrical tube for 2 h; (7) tail clamping (1 cm from the base of the tail) for 2 min; and (8) continuous inversion of the light/dark cycle for 24 h.

To ensure the stressors remained entirely unpredictable and to prevent habituation, one or two stressors were applied daily in a completely randomized sequence. Acute interventions, for example, tail clamping, restraint, or ice-water swimming were applied in isolation during the standard active phase, whereas continuous environmental interventions like damp bedding, fasting, or light-dark inversion spanned the full 24-h cycle. Crucially, the identical stressor was never applied on consecutive days and was separated by an interval of at least 7 days to maintain absolute unpredictability throughout the 3-week modeling phase.

**Figure 1:**
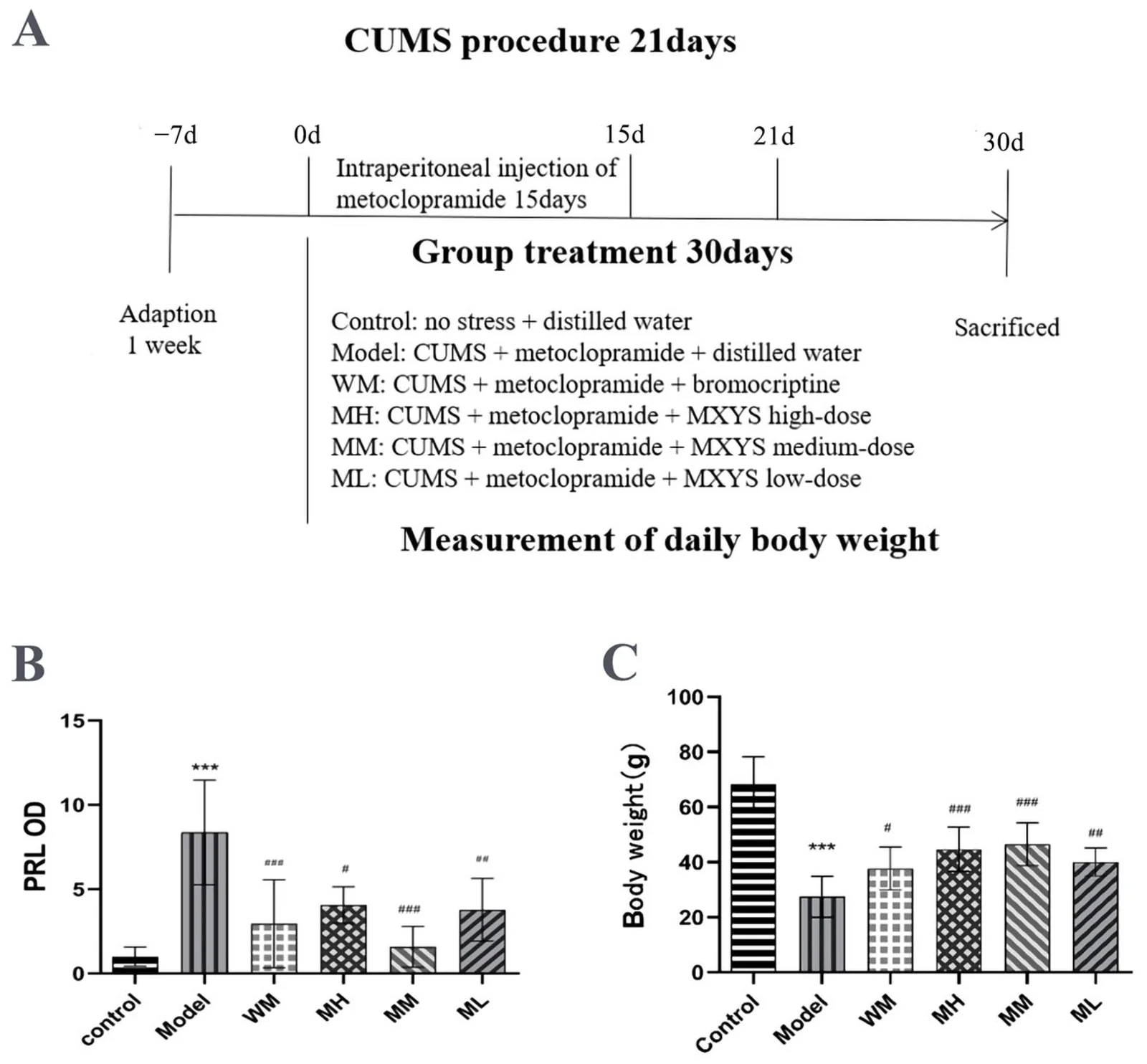
Effect of MXYS on PRL levels and body weight in the HPRL rat model. (**A**) Detailed experimental flowchart. Following 7 days of acclimatization, CUMS and metoclopramide (75 mg/kg, twice daily) interventions were administered. Treatments were given concurrently via daily gavage for 30 days. (**B**) Relative serum Prolactin (PRL) levels represented by Optical Density (OD) values (n = 12 per group). (**C**) Continuous body weight tracking recorded daily across the experimental period (n = 20 per group). Data are expressed as mean ± SD. Statistical differences for PRL were assessed using One-Way ANOVA with Tukey’s post hoc test, and body weight via Repeated Measures ANOVA. *** *p* < 0.001 vs. the Control group; # *p* < 0.05, ## *p* < 0.01, ### *p* < 0.001 vs. the Model group. MXYS, Modified Xiaoyaosan; WM, bromocriptine group; MH, high-dose MXYS; MM, medium-dose MXYS; ML, low-dose MXYS.

As illustrated in the experimental flowchart (Figure 1A), after 1 week of acclimatization (Days −7 to 0), the CUMS protocol and drug interventions were initiated simultaneously. From Day 0 to Day 15, rats received an intraperitoneal injection of metoclopramide hydrochloride (75 mg/kg twice daily). The CUMS procedure was conducted concurrently for 21 days (Days 0 to 21). Group treatments were administered daily via oral gavage for a total of 30 days (Days 0 to 30). Because different molecular assays require distinct tissue preparation methods that fresh frozen tissue for WB/PCR versus perfusion-fixed tissue for histology, the total cohort (n = 20 per group) was randomized prior to sacrifice using a computer-generated random number sequence to eliminate selection bias. Specifically, serum was collected from a subset of animals (n = 12) for ELISA, while hypothalamic tissues from another randomly selected subset (n = 6) were utilized for Western blot, Real-Time PCR, and immunohistochemical assays. Body weight was continuously tracked for all surviving animals (n = 20).

#### 2.2.2. Preparation of Drugs

The experimental formulation utilized in this study was Modified Xiaoyaosan (MXYS). MXYS builds upon the classic XYS prescription by incorporating Hordei Fructus Germinatus, a targeted botanical addition widely utilized in clinical practice to reduce abnormal prolactin secretion. The MXYS decoction comprises seven botanical drugs: Hordei Fructus Germinatus (*Hordeum vulgare* L; Poaceae) 30 g, Paeoniae Radix Alba (*Paeonia lactiflora* Pall; Paeoniaceae) 20 g, Bupleuri Radix (*Bupleurum chinense* DC; Apiaceae) 15 g, Angelicae Sinensis Radix (*Angelica sinensis* (Oliv.) Diels; Apiaceae) 15 g, Poria (*Poria cocos* (Schw.) Wolf; Polyporaceae) 15 g, Atractylodis Macrocephalae Rhizome (*Atractylodes macrocephala* Koidz; Asteraceae) 15 g, and Glycyrrhizae Radix et Rhizome (*Glycyrrhiza glabra* L; Fabaceae) 10 g. The fingerprint library from the Key Laboratory of Natural Products Chemistry, Chinese Academy of Sciences, Guizhou Province, was used to compare the constituents of MXYS, confirming that the primary active anti-inflammatory ingredients of the classic XYS formulation remain structurally dominant. These botanical drugs are mixed and steeped according to the TCM decoction method. To prepare MXYS, 120 g of the botanical drug mixture was soaked in distilled water (960 mL) for 30 min. The amount of distilled water (960 mL) was eight times the weight of the botanical drugs, and after boiling in distilled water for 30 min, the filtrate was filtered to obtain about 160 mL, and then the above operation was repeated for the second extraction. The combined filtrates (320 mL) were concentrated to 50 mL (2.4 g of crude herb/mL). The concentrated MXYS extraction was diluted to 0.6 g/mL and 1.2 g/mL, respectively, and stored at −80 °C. The fingerprint library from the Key Laboratory of Natural Products Chemistry, Chinese Academy of Sciences, Guizhou Province was used to compare the constituents of MXYS, which were found to contain several active ingredients, as detailed in the Supplemental Material. XYS phytoconstituents were identified using UPLC-MS/MS (Ultimate 3000 UPLC coupled to a Q Exactive Focus MS system, ThermoFisher Scientific, Waltham, MA, USA). Our prescription for MXYS by the Pharmacy Department of the First Affiliated Hospital of Guizhou University of TCM. All formulation components met the requirements of the relevant items of the People’s Republic of China Pharmacopoeia in 2015. Bromocriptine mesylate (2.5 mg/tablet, 30 tablets/bottle) Novartis Farma S.p.A., Origgio, Italy; batch number: T87612A) was prepared as a 0.1 mg/mL suspension with distilled water and set aside. Metoclopramide hydrochloride injection (specification: 1 mL: 10 mg) (Shanghai Hefeng, Shanghai, China; H31021522) was also used.

The HPRL rat model was developed by administering metoclopramide hydrochloride intraperitoneally, in combination with CUMS. The MXYS extract was administered at a volume of 1 mL/100 g body weight. Based on the extract concentrations, the final oral doses for the high-, medium-, and low-dose MXYS groups were equivalent to 24, 12, and 6 g/kg/day of raw crude herbs, respectively. After 7 days of acclimatization feeding, each group was modeled by intraperitoneal injection of metoclopramide hydrochloride at 75 mg/kg twice daily over 15 days, except for the control group. CUMS was synchronized with drug intervention, and CUMS was induced for 21 days. Starting drug administration at the end of modeling, 14 days of treatment by gavage, the bromocriptine group (1 mg/kg/d) and the MXYS high, medium, and low-dose groups (2.4, 1.2, and 0.6g/mL, respectively) were administered through gavage, equivalent to 1, 2, and 4 times the clinical equivalent dose. The MXYS extract was administered at a dose of 1 mL/100 g. The rat dosage of MXYS was calculated using a human-to-animal dose conversion formula, with a conversion factor of 6. The medium dose of MXYS for rats was obtained by using the following formula: medium dose of XYS = 120 g (the total amount of raw materials)/60 kg (normal human body weight) × 6 (conversion factor). After the final gavage administration, the animals were fasted overnight. Serum was collected from the abdominal aorta after intraperitoneal injection anesthesia with 3% pentobarbital sodium, which ensured that the animals were fully anesthetized. Rats were euthanized via cervical dislocation, and the hypothalamus was extracted under sterile conditions and stored at −80 °C for Western blotting (WB), PCR, Immunofluorescence staining, and immunohistochemical assays. To evaluate whether the modeling and therapy processes affected the weight changes of the rats, their body weight was measured and recorded in each group at 8 a.m. from day 1 until the end of the experiment. The timeline is shown in Figure 1A.

#### 2.2.3. ELISA

The rats were anesthetized with pentobarbital after the final dose, and blood samples were taken from the abdominal aorta to extract serum. The serum prolactin levels were determined using an ELISA kit (Elite, Wuhan, Beijing) and the manufacturer’s instructions.

#### 2.2.4. Immunohistochemistry

Following standard protocols, immobilized tissues were dehydrated, embedded, and sectioned using an automated dehydrator. Then, the slices were incubated with primary antibodies (TH, TNF-α, and IBA-1 from Abcam at 1:100 dilution; GFAP from Boossen, Beijing, China at 1:200 dilution; and IL-1β from PROTEINTECH at 1:50 dilution) overnight at 4 °C. Subsequently, the sections at 37 °C to incubate 30 min the secondary antibodies, followed by color development with DAB (Beijing, China), hematoxylin and eosin staining, dehydration, and sealing. Positive staining was identified by brownish-yellow cytoplasmic membrane staining, and a negative control was used. Images of the sections were captured using the BA200 digital trinocular microscope (Motic, Xiamen, China) camera system and analyzed with Image-Pro Plus 6.0 image analysis software (Media Cybernetics, Rockville, MD, USA). The mean optical intensity of each section was calculated under the microscope.

#### 2.2.5. WB Assay

The entire protein was extracted using RIPA cell lysis buffer. After centrifugation at 10,000 rpm for 20 min at 4 °C, the protein concentration was measured using the BCA kit (E-BC-K318-M, Elabscience (Wuhan, China)). The SDS-PAGE loading buffer was combined with an equal quantity of protein extract. Electrophoresis on an 8–10% SDS-PAGE gel separated the total protein, which was then transferred to PVDF membranes. The membranes were blocked with 5% non-fat dry milk in TBST for 1 h at room temperature to prevent non-specific binding. Subsequently, the membranes were incubated at 4 °C overnight with primary antibodies (iNOS and NOX2 from Abcam (Hangzhou, China); Drd2 from Thermo (Waltham, MA, USA); and GAPDH as a loading control, 1:1000). The membranes were washed three times with TBST for 10 min each, incubated with HRP-conjugated secondary antibodies (1:5000) for 1 h at room temperature, rinsed again, and subjected to color development with DAB. Finally, the membrane was treated with ECL (Roche Diagnostics, Penzberg, Germany), and the results were analyzed using the accompanying software.

#### 2.2.6. Real-Time-PCR

The total mRNA from the hypothalamus was extracted using an isolation kit (NO: RE-03014, FOREGENE (Chengdu, China)) following the manufacturer’s instructions. The cDNA was generated with the RT Easy II kit (NO: RT-01023, FOREGENE (Chengdu, China)) reverse transcription kit. The primer for Cryab was designed and synthesized by Shanghai Bioengineering Biotechnology Service Co. (Shanghai, China) (Forward primer: TTCGGAGAGCACCTGTTGGAGTCTGA; Reverse primer: ACGCATCTCTGAGAGCCCAGTGTCAA). PCR criteria were set at 95 °C (30 s pre-denaturation), 95 °C (5 s denaturation), 55 °C (30 s annealing), 72 °C (30 s extension), and 45 cycles. Detecting the CT (threshold cycle) values during PCR by Thermo Scientific PikoReal software v13.0 (Thermo Fisher, Waltham, MA, USA) per sample, Cryab mRNA expression was normalized to the endogenous reference gene GAPDH and quantified using the 2^−ΔΔCT^ method.

#### 2.2.7. Immunofluorescence Staining

According to the experimental reagent directions. After embedding and baking the paraffin sections, they were dewaxed, hydrated, and repaired with antigen. Heated for 5 min, chilled for 10 min, then heated again for 5 min. After cooling, the antigen repair solution was discarded, and the sections were rinsed with PBS. The sections were permeabilized after 20 min of incubation at room temperature with 0.5% TritonX-100 in PBS (Foconsci Chemical Industry Co., Ltd., Zaozhuang, China), followed by three PBS washes lasting 5 min each. 5% BSA was applied to the slides and incubated for 30 min at 37 °C to prevent non-specific binding. The primary antibodies (GFAP, PROTEINTECH, 1:100; IBA-1, Affinity, 1:100; TH, Affinity, 1:200 (While Proteintech, Wuhan, China)) were diluted and added dropwise to the sections, which were then incubated overnight at 4 °C in a wet box. Subsequently, the sections were incubated with the secondary antibody (cy3, ABclonal, 1:100 (Wuhan ABclonal Biotechnology Co., Ltd., Wuhan, China)) at 37 °C for 45 min. Finally, the sections were stained with DAPI and sealed.

#### 2.2.8. Experimental Statistical Analysis

Data are presented as the mean and standard deviation (SD) from three separate experiments. All outcome assessments, including the quantification of immunohistochemistry, immunofluorescence, and behavioral data, were performed by independent investigators who were strictly blinded to the experimental group assignments in order to eliminate observer bias. Statistical analysis was conducted using SPSS 25.0 and GraphPad Prism 9. For experimental comparisons involving multiple independent groups at a single time point, a One-Way Analysis of Variance (ANOVA) followed by Tukey’s post hoc test was utilized for ELISA, immunohistochemistry, Western blot, and Real-Time PCR data. A Repeated Measures ANOVA was applied to evaluate the changes in body weight over time across the six experimental groups. *p* < 0.05, statistical significance was taken into account. Details were shown in Supplementary Materials.

## 3. Results

### 3.1. Phase 1 Meta-Analysis: Xys Improves Behavioral Despair and Anhedonia in Cums Models

**Figure 2:**
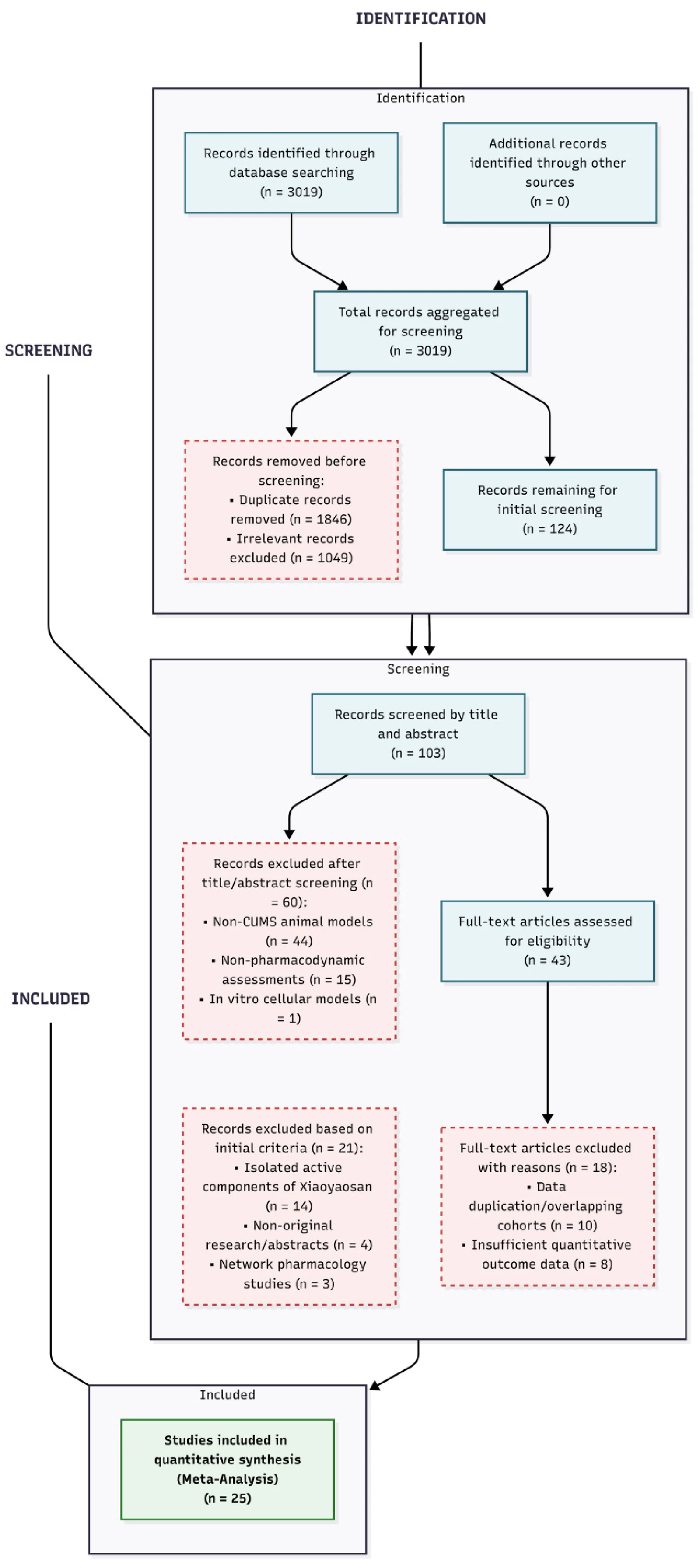
PRISMA flowchart of the literature inclusion process for the preclinical meta-analysis. The flowchart details the identification, screening, and inclusion phases yielding the final 25 studies evaluated.

The meta-analysis evaluated 25 studies involving 569 animals to assess the broad efficacy of XYS on CUMS-induced behavioral deficits [12,13,14,15,16,17,18,19,20,21,22,23,24,25,26,27,28,29,30,31,32,33,34,35,36] (Figure 2 and Figures S1 and S2, Table 1). The detailed results of research characteristics and quality evaluation were shown in Supplementary Results. As for body weight and food intake, CUMS significantly reduced body weight and appetite in the model groups (*p* < 0.001). Treatment with XYS effectively reversed these physical symptoms, significantly increasing body weight (n = 21; SMD = 2.65, 95% CI: 2.13–3.17, I^2^ = 0.751) and restoring food intake (n = 11; SMD = 4.88, 95% CI: 3.62–6.13, I^2^ = 0.876) (Figures S3 and S4). The detailed analysis results of body weight and food intake were shown in Supplementary Results.

In the sucrose preference test (SPT), XYS significantly increased sucrose consumption, indicating a restoration of pleasure-seeking behavior (n = 24; SMD = 3.43, 95% CI: 2.62–4.23, I^2^ = 0.899, *p* < 0.001) (Figure S5). Furthermore, the forced swimming test (FST) demonstrated that XYS significantly reduced immobility time, ameliorating behavioral despair (n = 9; SMD = −4.97, 95% CI: −6.29–−3.65, I^2^ = 0.820 (Figures S6 and S7). Moreover, to investigate exploratory behavior, the Open field test (OFT) results revealed that XYS significantly increased the total running distance (n = 14; SMD = 3.96, 95% CI: 2.90–5.02, I^2^ = 0.894), the number of entries into the central zone, and the number of upright times, successfully relieving tension and restoring the animals’ desire to explore new environments (all *p* < 0.001) (*p* < 0.001) (Figures S8–S10). The detailed SPT, FST, and OFT analysis results were shown in Supplementary Results. To assess potential publication bias, Egger’s test was performed on two primary outcomes: body weight and the SPT. The findings indicated the presence of statistically significant publication bias across the included studies for both body weight (*p* = 0.002) and SPT (*p* < 0.001) (Figures S11–S13).

Given the high heterogeneity observed across the pooled data, subgroup analyses were conducted to stratify the primary outcomes, including body weight and SPT, by dosage, animal species, and administration duration (Figures S12 and S13). For both body weight and SPT, stratification revealed that XYS dosage was the primary source of heterogeneity, and variance was significantly reduced in the medium-dose and high-dose cohorts, while remaining elevated in the low-dose groups. Stratification by animal species (rats vs. mice) yielded no significant difference in the pooled effects for either outcome, confirming cross-species efficacy. Finally, stratifying by administration time demonstrated that a 28-day intervention optimally reduced heterogeneity for both parameters. Despite these stratifications resolving variance for primary outcomes, the heterogeneity for secondary behavioral assessments for OFT and food intake remained high, indicating that variations in experimental design heavily influence these specific indices.

### 3.2. Phase 2 Physiological Outcomes: Xys Ameliorates Weight Loss and Reduces Prl Levels in Hprl Rats

Building upon the weight-protective effects observed in the meta-analysis, our in vivo experiment confirmed that CUMS combined with metoclopramide induced severe weight loss in HPRL rats. Both XYS and bromocriptine exhibited protective effects against this weight loss, with XYS demonstrating superior efficacy compared to bromocriptine.

Regarding endocrine regulation, the HPRL model group exhibited significantly elevated serum prolactin (PRL) levels (8 ng/mL, mean OD value = 8.3) compared to the control group (1 ng/mL, mean OD value = 1.0) (*p* < 0.001). Treatment with XYS effectively reversed this abnormal secretion, significantly reducing PRL levels to 2 ng/mL (mean OD value = 8.3), which was comparable to the therapeutic effect of the clinical standard bromocriptine (3 ng/mL, mean OD value = 2.9) (Figure 1B).

HPRL model group rats lost markedly more weight than the control group, and as shown in Figure 1C, both XYS and bromocriptine were found to have protective effects against weight loss, with XYS showing better efficacy than bromocriptine. These findings indicate that XYS can reduce PRL levels and ameliorate weight loss in HPRL rats, suggesting that XYS may have therapeutic potential for treating HPRL.

### 3.3. Cellular Mechanisms: Xys Inhibits Microglia and Astrocyte Activation to Protect Dopaminergic Neurons

To elucidate the cellular mechanisms driving these systemic improvements, we evaluated the inflammatory states of key immunomodulatory cells in the central nervous system (CNS).

**Figure 3:**
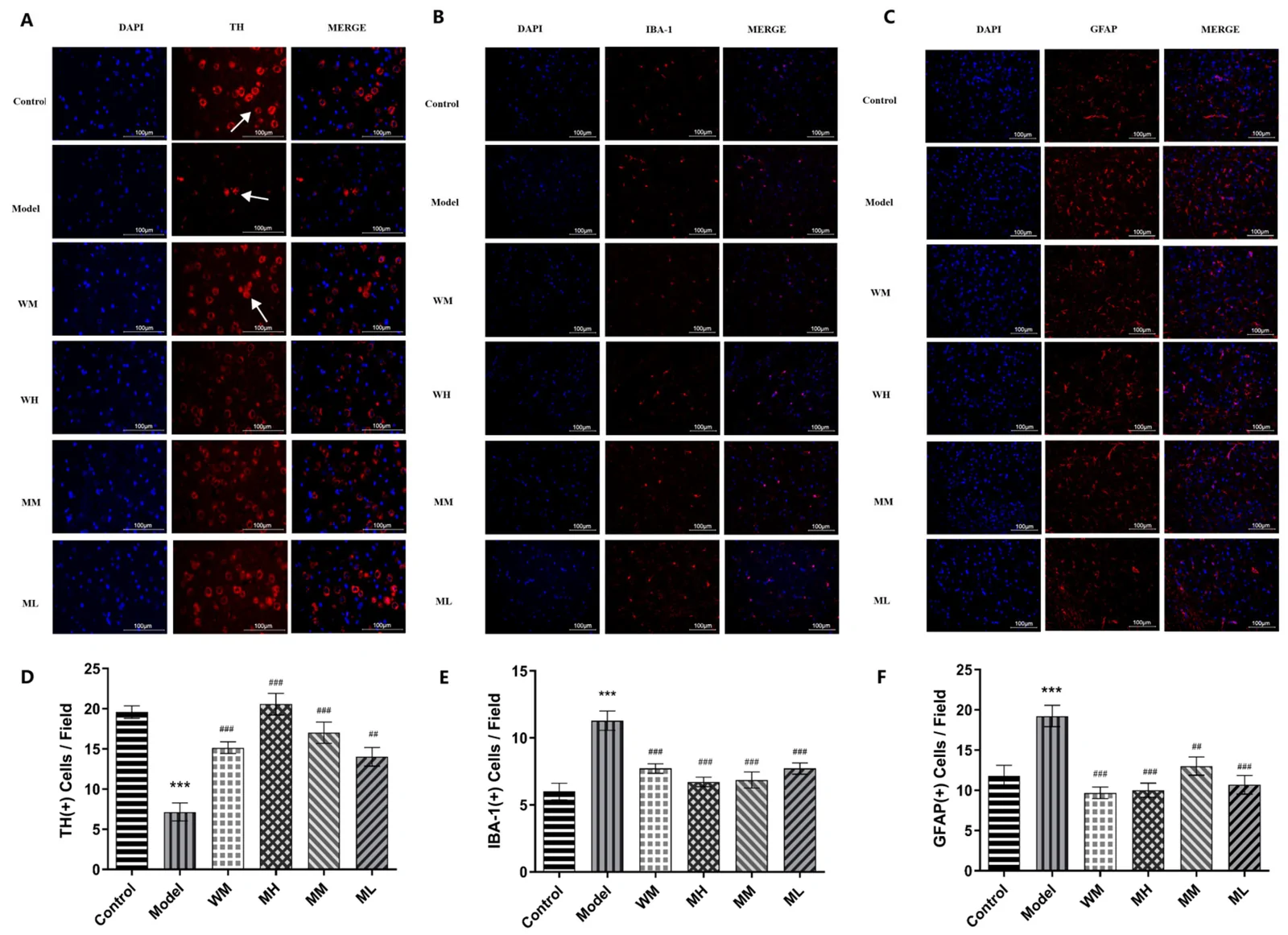
Effects of MXYS on localized glial response markers and TH-positive expression in the rat hypothalamus. (**A**–**C**) Representative immunofluorescence staining images for Tyrosine hydroxylase (TH, a specific marker for dopaminergic neurons), Ionized calcium-binding adapter molecule 1 (IBA-1, a microglial marker), and Glial fibrillary acidic protein (GFAP, an astrocyte marker). Scale bar = 20 µm. (**D**–**F**) Quantitative analysis of the mean fluorescence intensity for TH, IBA-1, and GFAP (n = 6 per group). Data are expressed as mean ± SD. Statistical differences were assessed using One-Way ANOVA followed by Tukey’s post hoc test. *** *p* < 0.001 vs. the Control group; ## *p* < 0.01, ### *p* < 0.001 vs. the Model group. Note: The white arrows indicated TH-positive neurons for immunofluorescence.

Immunofluorescence assays revealed that the model group exhibited elevated fluorescence intensities of their respective glial biomarker proteins, IBA-1 (microglia) and GFAP (astrocytes). XYS across all three dose groups significantly reduced IBA-1 and GFAP expression (Figure 3B–F). While these marker changes do not fully demonstrate functional activation states, they suggest that XYS successfully attenuates localized glial responses to stress and pharmacological challenge.

Tyrosine hydroxylase (TH) serves as the rate-limiting enzyme and specific marker for the dopaminergic phenotype in the hypothalamus. Both immunofluorescence and immunohistochemistry results demonstrated that XYS significantly augmented TH immunoreactivity and increased the number of cells with detectable TH expression compared to the model group (Figure 3A,D). While increased TH expression did not directly prove absolute neuronal survival or the prevention of apoptosis without further viability assessments such as TUNEL, it indicated that the attenuation of surrounding glial responses correlated with the restoration of the dopaminergic phenotype.

### 3.4. Molecular Mechanisms: Xys Regulates the Nf-Κb Pathway and Cryab to Suppress Inflammation

Further mechanistic investigations were conducted to map the specific inflammatory pathways modulated by XYS.

**Figure 4:**
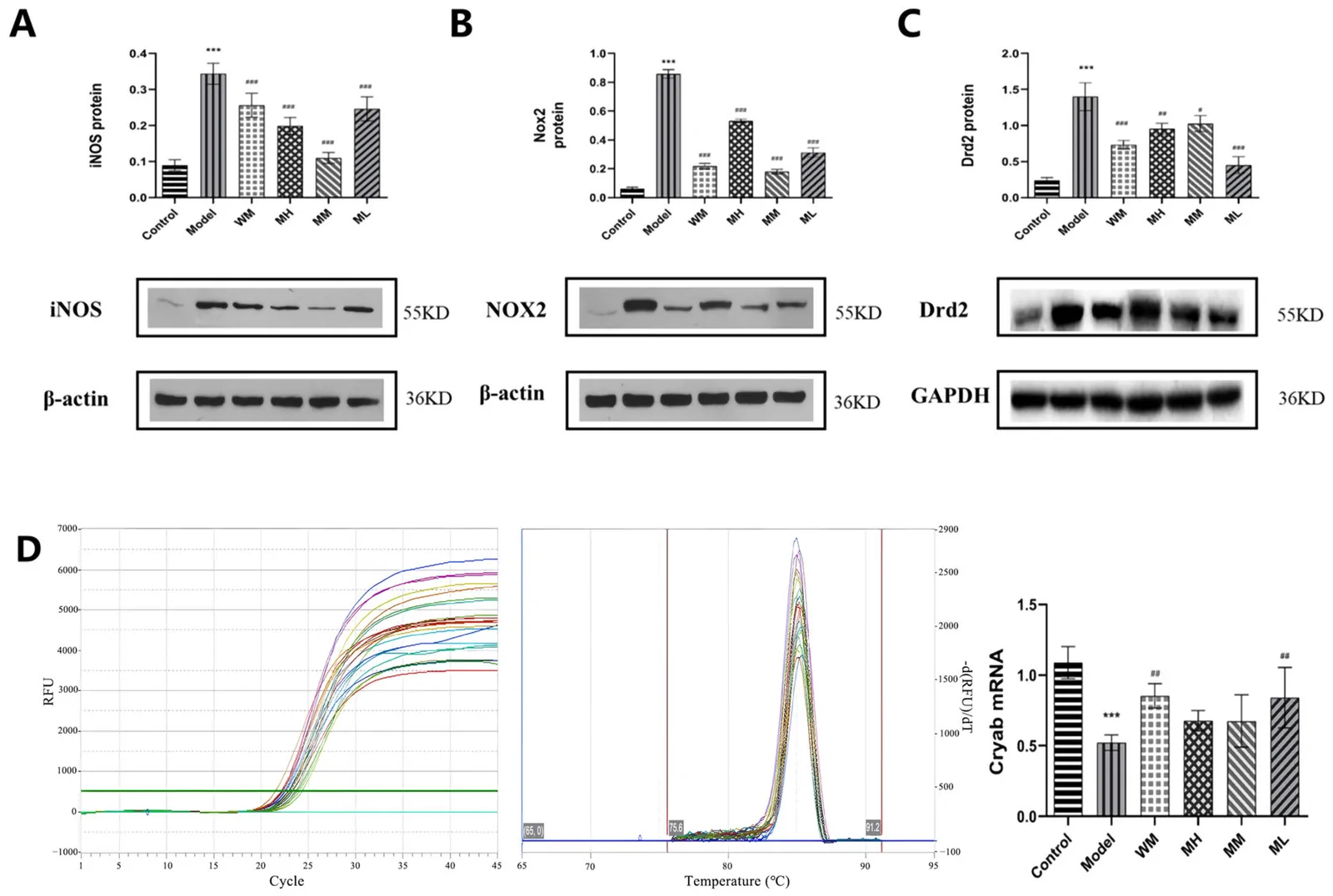
MXYS modulates the expression of upstream NF-κB inflammatory cascade proteins and Drd2 in the hypothalamus. (**A**–**C**) Representative Western blot images and quantitative densitometric analysis for iNOS, NOX2, and Dopamine D2 receptor (Drd2) normalized to GAPDH (n = 6 per group). (**D**) Relative mRNA expression levels of the regulatory protein Cryab assessed via Real-Time PCR, normalized to GAPDH (n = 6 per group). Data are expressed as mean ± SD. Statistical differences were assessed using One-Way ANOVA followed by Tukey’s post hoc test. *** *p* < 0.001 vs. the Control group; # *p* < 0.05, ## *p* < 0.01, ### *p* < 0.001 vs. the Model group. iNOS, inducible nitric oxide synthase; NOX2, NADPH-Oxidase 2.

Microglial alterations often target NOX2, which produces iNOS to trigger the CNS inflammatory response via downstream pathways, including NF-κB. Western blot analysis showed that iNOS, NOX2, and Dopamine D2 receptor (Drd2) were significantly elevated in the HPRL model group. XYS significantly reduced the expression of iNOS, NOX2, and Drd2, effectively blunting this upstream inflammatory cascade (Figure 4A–C).

**Figure 5:**
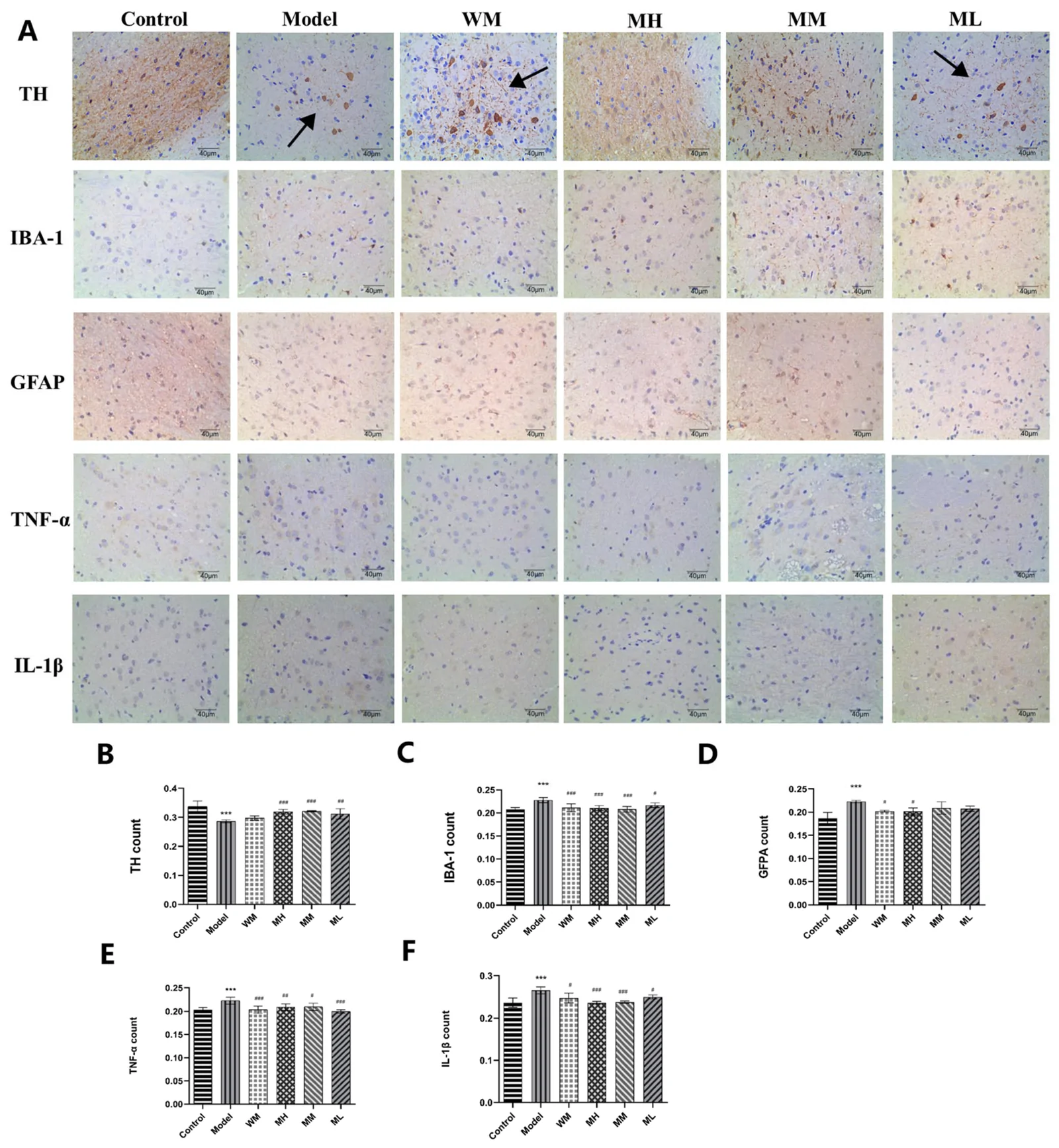
MXYS attenuates the downstream expression of pro-inflammatory cytokines in the rat hypothalamus. (**A**) Representative immunohistochemistry (IHC) staining images evaluating TH, IBA-1, GFAP, TNF-α, and IL-1β. Note: The black arrows indicated TH-positive neurons for Immunohistochemistry. (**B**–**F**) Quantitative analysis of the mean optical density for the respective IHC markers (n = 6 per group). Data are expressed as mean ± SD. Statistical differences were assessed using One-Way ANOVA followed by Tukey’s post hoc test. *** *p* < 0.001 vs. the Control group; # *p* < 0.05, ## *p* < 0.01, ### *p* < 0.001 vs. the Model group. TNF-α, Tumor necrosis factor alpha; IL-1β, Interleukin-1 beta. Note: (**A**) displays the immunohistochemistry results of TH (dopaminergic neurons), IBA-1 (microglia), GFAP (astrocytes), and pro-inflammatory factors TNF-α and IL-1β in HPRL rats. (**B**) shows that TH levels in all three XYS dose groups were dramatically increased over the model group. (**C**,**D**) demonstrate that both bromocriptine and XYS can reduce IBA-1 and GFAP expression, which in turn reduces microglia and astrocyte hyperactivation. (**E**,**F**) show that bromocriptine and XYS can significantly reduce the expression of inflammatory factors TNF-α and IL-1β compared to the model group.

Cryab is a crucial target protein associated with NF-κB signaling that helps regulate the secretion of anti-inflammatory factors. Real-Time PCR demonstrated that XYS significantly upregulated Cryab mRNA levels (Figure 4D). Consequently, XYS significantly reduced the downstream secretion of pro-inflammatory factors, specifically TNF-α and IL-1β, as confirmed by immunohistochemistry (Figure 5). Although our study did not directly measure NF-κB nuclear translocation or phosphorylation, the concurrent modulation of these related upstream (NOX2/iNOS) and regulatory (Cryab) targets suggests XYS effectively buffered the broader neuro-inflammatory network.

## 4. Discussion

Negative chronic stress is an established pathogenic factor for systemic wasting diseases, including depression and HPRL. Our dual-evidence approach—combining a comprehensive meta-analysis of CUMS-induced depression with an in vivo exploration of CUMS-induced HPRL—reveals that the diverse physical and behavioral symptoms of chronic stress share underlying neuro-immunological origins. Our meta-analysis confirmed that XYS significantly ameliorates stress-induced weight loss, anhedonia, and behavioral despair. Corroborating this, our experimental data demonstrated a significant negative correlation between PRL levels and body weight, indicating that CUMS-induced endocrine disruption leads directly to physical wasting in HPRL. Most importantly, our findings suggest that XYS reverses these diverse pathologies through a unified, synergistic mechanism: modulating the neuro-immune microenvironment to protect neuronal integrity.

**Figure 6:**
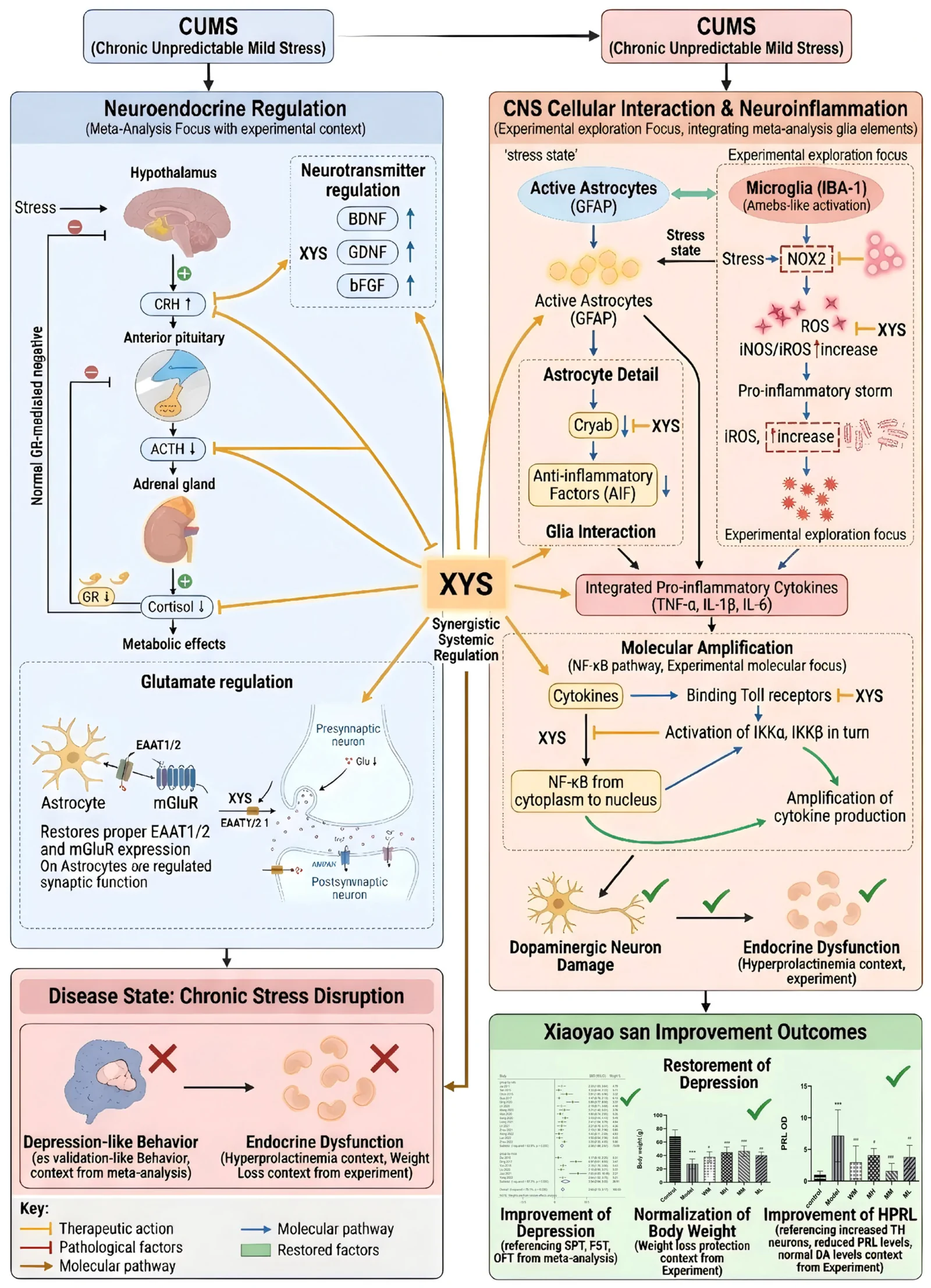
Proposed theoretical schema bridging the meta-analytical and experimental findings. A visual representation summarizing how classic Xiaoyaosan (XYS) and Modified Xiaoyaosan (MXYS) synergistically buffer the systemic neuro-immune microenvironment. The diagram maps the neuroendocrine regulation (identified via meta-analysis) and the attenuation of localized neuroinflammation and glial responses (explored via the in vivo HPRL experiment). *** *p* < 0.001, # *p* < 0.05, ## *p* < 0.01, ### *p* < 0.001.

Chronic stress induces systemic inflammation, which fundamentally alters mood, cognition, and endocrine balance. The meta-analysis established that XYS effectively exerts antidepressant effects by reducing the expression of pro-inflammatory cytokines, specifically IL-6 and TNF-α, in the hippocampus and serum [37]. Our experimental study has provided a molecular context for this anti-inflammatory effect: XYS targets proteins integral to the NF-κB signaling cascade. By downregulating NOX2 and iNOS, XYS successfully limited the secretion of pro-inflammatory factors, including TNF-α and IL-1β. Furthermore, our study uniquely identified that XYS upregulates Cryab, a crucial modulatory protein to promote anti-inflammatory responses. Our study found that XYS significantly increased Cryab mRNA at low doses, demonstrating its therapeutic effect similar to that of bromocriptine. Mechanistic studies confirmed that XYS could synergistically target multiple molecules of the NF-κB pathway, improve the expression levels of molecules associated with the reversal of inflammation, inhibit the inflammatory response, and ultimately protect dopaminergic neurons and promote DA expression. The potential mechanism of action of XYS explored by experiments has been illustrated in Figure 6.

Astrocytes and microglia are the primary immunomodulatory cells in the CNS and play a critical role in the cyclic inflammatory responses mediated by the NF-κB pathway. The meta-analysis highlights that stress-induced astrocyte dysfunction leads to an overactive glutamate system, a primary driver of depression. XYS regulates astrocyte function, upregulates glial fibrillary acidic protein (GFAP), and promotes glutamate clearance [38]. Additionally, expanding on this, our experimental data proved that XYS successfully inhibits the hyperactivation of both astrocytes (GFAP) and microglia (IBA-1). By reducing this hyperactivation, XYS effectively halts the toxic cycle of neuroinflammation at its cellular source.

Ultimately, the regulation of neuroinflammation and glial cells serves to protect the brain’s delicate neuro-endocrine networks. The meta-analysis demonstrated that XYS regulates the HPA axis by downregulating adrenocorticotropic hormone (ACTH) and cortisol/corticosterone (CORT) levels, while simultaneously increasing neuroprotective proteins like brain-derived neurotrophic factor (BDNF) [13,39]. Otherwise, in the context of HPRL, abnormal PRL secretion is intimately tied to the functional suppression of dopaminergic signaling in the hypothalamus. Our experimental results revealed that by improving this local immune microenvironment, XYS restored the dopaminergic phenotype. While we could not definitively claim direct neuronal survival, this preservation has been evidenced by increased TH levels and a reduction in the compensatory increase in Drd2. Consequently, this neuronal protection upregulates DA levels, which directly inhibits pituitary PRL secretion and resolves the HPRL pathology.

The implications of this dopaminergic preservation have extended well beyond the immediate regulation of endocrine and mood disorders. Chronic stress is known to profoundly alter dopaminergic transmission by impacting dopamine synthesis, release, and receptor sensitivity through the exacerbation of neuroinflammation and microglial activation [40,41,42]. This stress-induced vulnerability within the dopaminergic system is a well-established mechanism, contributing heavily to the pathogenesis of neurodegenerative disorders, most notably Parkinson’s disease [40]. Because MXYS effectively attenuated this precise localized microglial response (IBA-1) and modulated the secondary receptor apparatus (Drd2), it was highly plausible that the formulation functions as a broad non-specific neuroprotectant. MXYS might not only resolve acute systemic disruptions but also reduce the long-term likelihood of progressive dopaminergic neurodegeneration through chronically buffering the neuro-immune microenvironment against stress-induced inflammatory cascades.

A critical synthesis of the meta-analytical and experimental data revealed that the behavioral recovery observed in depression models and the endocrine recovery seen in HPRL rats were overlapping phenomena influenced by a shared immunomodulatory network. Chronic stress has triggered systemic inflammation, acting as a central pathogenic hub with disruption of mood, cognition, and neuroendocrine balance. By comparing the two datasets, it became evident that XYS acted on this shared pathogenic network to provide broad systemic buffering, even when distinct localized pathologies were present (Table 2).

In our experimental model, bromocriptine was utilized strictly as a positive phenotypic control to benchmark PRL reduction through direct D2 receptor agonism. In contrast, MXYS did not function as a direct receptor agonist, whereas it indirectly modulated the endocrine system by attenuating upstream inflammatory pathways. Therefore, these interventions represented fundamentally distinct pharmacological mechanisms, highlighting MXYS as a systemic neuro-immune buffer rather than a direct endocrine targeted therapy. By comparing the two datasets, it becomes evident that MXYS inherits and leverages the shared pathogenic network targeted by classic XYS. Because the core chemical constituents are conserved between the formulations, the robust systemic anti-inflammatory effects of XYS established by the meta-analysis provide the mechanistic foundation for MXYS. The addition of Hordei Fructus Germinatus in MXYS optimizes this foundational neuro-immune buffering capacity specifically for an endocrine challenge. The meta-analysis highlights that stress-induced astrocyte dysfunction is central to the pathogenesis of depression, and XYS effectively regulates astrocyte activity, as indicated by changes in the expression of GFAP. Our in vivo experiment corroborates and expands upon this mechanism, demonstrating that XYS prevents the hyperactivation of both astrocytes (GFAP) and microglia (IBA-1), which serve as the primary immunomodulatory cells in the CNS.

The meta-analysis confirms that XYS exerts antidepressant effects by reducing widespread inflammatory responses, notably lowering TNF-α and IL-6 levels in the hippocampus and serum of depressed rodents. Our experimental exploration mirrors this anti-inflammatory outcome but traces it to a specific molecular origin: XYS directly modulates the NF-κB pathway. By downregulating the critical targets NOX2 and iNOS, and upregulating Cryab, XYS directly inhibits the secretion of TNF-α and IL-1β. Additionally, in the CUMS-induced depression model analyzed in the meta-analysis, calming this inflammatory network restores the HPA axis, as evidenced by normalized ACTH and cortisol/CORT levels (Figure 6). Concurrently, in the CUMS-induced HPRL model, alleviating this exact same CNS inflammatory state protects hypothalamic dopaminergic neurons, upregulates DA levels, and suppresses pituitary PRL secretion.

To fully evaluate the translational value of MXYS, it is necessary to benchmark its efficacy against standard modern pharmacological interventions. Modern management of stress-induced depression relies heavily on Selective Serotonin Reuptake Inhibitors (SSRIs) such as fluoxetine, while hyperprolactinemia is standardly managed with dopamine D2-receptor antagonists like bromocriptine or cabergoline. While highly effective, these targeted monotherapies often present dose-limiting toxicities, treatment resistance, or adverse metabolic side effects. Independent comparative studies have demonstrated that while classic XYS might exhibit a slower onset of action compared to SSRIs in acute depressive episodes, it offers superior long-term stabilization of the neuro-immune microenvironment with a markedly lower adverse effect profile [12,31,43]. In our experimental model, MXYS demonstrated a capacity to reduce serum PRL levels and attenuate glial responses comparably to the clinical standard bromocriptine. Crucially, unlike isolated D2-antagonists, MXYS simultaneously suppressed broader systemic inflammatory cytokines (TNF-α and IL-1β) [10,,44]. This independent comparison highlights MXYS not as a replacement for targeted acute pharmacotherapy, but as a robust multi-target complementary strategy capable of managing the systemic inflammatory burden that often complicates chronic stress disorders.

This essential comparison argues that CUMS-induced behavioral despair and endocrine disruption are disparate symptoms of the same neuro-immune failure. By comprehensively targeting the NF-κB-mediated inflammatory cascade and resetting glial cell activation, XYS effectively repairs this core immunomodulatory network. In doing so, it simultaneously drives the behavioral recovery noted in the meta-analysis and the endocrine recovery proven in our experiment. While this dual-evidence approach has offered comprehensive insights, several limitations still exist. First, there was an inherent logical gap in extrapolating mechanisms from a meta-analysis of classic XYS to an in vivo experiment utilizing MXYS. Although MXYS retains the foundational botanical matrix of classic XYS, our experimental design lacked a head-to-head XYS control arm. Therefore, without a direct comparative analysis of their pharmacodynamic profiles, the assumption of equivalent bioactive mechanisms remained a methodological simplification. In addition, a notable limitation of this study was the absence of isolated “CUMS-only” and “metoclopramide-only” control groups, so the observed endocrine recovery and dopaminergic preservation could not be attributed exclusively to the reversal of chronic stress. Further experimental designs should incorporate isolated stress and pharmacological cohorts to definitively parse these variables. Consequently, the current study established the preventive potential of MXYS against a compounded pathology, rather than a curative intervention for established disease. Crucially, Egger’s test revealed a significant publication bias within the meta-analysis regarding two primary outcomes, including body weight (*p* = 0.002) and the SPT (*p* < 0.001). This bias suggested a potential overrepresentation of positive outcomes in the existing literature, which might artificially inflate the pooled effect sizes for XYS’s behavioral and physical benefits. This limitation might underscore the necessity of our subsequent in vivo experimental validation, which was essential to objectively corroborate the formulation’s physical protective effects and underlying neuro-immune mechanisms under controlled conditions. Furthermore, the high unresolvable heterogeneity (I^2^ > 80%) observed in secondary outcomes such as OFT and food intake indicated that these specific pooled effects should be interpreted with caution. These limitations might underscore that while MXYS acts as a powerful neuro-immune buffer, its targeted mechanisms require further isolated validation. Finally, while this study mapped a compelling systemic regulatory network, the specific molecular connections linking hypothalamic neuroinflammation to pituitary PRL secretion remained correlative. Because this study did not employ targeted functional experiments, such as specific receptor blockades, CRISPR-mediated gene knockdowns, or precise apoptosis assays, it remained a proposed hypothesis requiring future functional validation considering the exact causal chain bridging the CNS immune microenvironment to peripheral endocrine outcomes.

## 5. Conclusions

By integrating broad preclinical meta-analysis data with targeted in vivo experimentation, this study has innovatively demonstrated that XYS acts as a multi-target systemic regulator. Under conditions of chronic stress, XYS alleviates the CNS inflammatory state by synergistically targeting the NF-κB pathway and Cryab to inhibit microglia and astrocyte hyperactivation. This immunomodulation not only regulates the HPA axis and glutamate systems to resolve depressive behaviors, but also decisively protects dopaminergic neurons to upregulate DA and reduce PRL secretion in HPRL. These findings provide a comprehensive biological mechanism supporting the widespread clinical application of XYS for stress-induced systemic and endocrine disorders.

## Figures and Tables

**Table 1:** Characteristics of included preclinical studies in the Meta-analysis.

Studies	Species (E/M)	Sex	Model	Anesthetic	Dose (g/kg)	Course/Day	Outcome Index
Dai et al. (2010) [14]	SD (8/8)	Male	CUMS	NM	13.50; 6.75; 3.38	28	BW; SPT; OFT
Jia et al. (2011) [15]	SD (8/8)	Male	CUMS	NM	92.40; 46.20; 23.10	28	BW; SPT; OFT
Tan et al. (2015) [16]	Wistar (12/12)	Male	CUMS	NM	19	21	BW; FI; SPT; OFT
Chen et al. (2015) [17]	SD (6/6)	Male	CUMS	NM	46.2	28	BW; SPT; OFT
Ding et al. (2017) [18]	C57BL/6J (15/15)	Male	CUMS	pentobarbital	0.25	21	BW; SPT; OFT
Guo et al. (2017) [19]	SD (10/10)	Male	CUMS	10% Chloral hydrate	0.197	21	BW; SPT; NFS; OFT
Yan et al. (2018) [13]	C57BL/6J (15/15)	Male	CUMS	pentobarbital	0.25	21	BW; FI; SPT; OFT
Ma et al. (2019) [20]	C57BL/6J (15/15)	Male	CUMS	pentobarbital	0.25	21	SPT; FST; OFT
Xu et al. (2019) [21]	SD (12/12)	Male	CUMS	NM	19.27	21	SPT
Lv et al. (2020) [22]	SD (6/6)	Male	CUMS	NM	46.3	28	BW; SPT; OFT
Liu et al. (2020) [23]	C57BL/6J (10/10)	Male	CUMS	NM	0.265	21	BW; FI; SPT; FST
Wang et al. (2020) [24]	SD (6/6)	Male	CUMS	NM	46.3; 23.1	28	BW; SPT; FST; OFT
Wan et al. (2020) [25]	SD (10/10)	Male	CUMS	10% Chloral hydrate	3.854	49	BW; FI; SPT; OFT
Ding et al. (2020) [26]	SD (10/10)	Male	CUMS	NM	2.224	42	BW; FI; SPT; OFT
Song et al. (2020) [12]	SD (20/20)	Male	CUMS	NM	2.224	42	BW; FI; SPT; FST; OFT
Yan et al. (2021) [27]	C57BL/6J (15/15)	Male	CUMS	beheaded	0.254	21	SPT; FST; NFS
Lv et al. (2021) [28]	SD (6/6)	Male	CUMS	NM	46.3	28	BW; SPT; FST; OFT
Zhou et al. (2021) [29]	SD (18/18)	Male	CUMS	pentobarbital	2.224	21	BW; FI; SPT; FST; OFT
Liang et al. (2021) [30]	SD (7/8)	Male	CUMS	pentobarbital	2.224	28	BW; SPT; NFS
Jiao et al. (2021) [31]	C57BL/6J (12/12)	Male	CUMS	pentobarbital	0.254	21	BW; FI; SPT; FST; NFS; OFT
Yuan et al. (2022) [32]	SD (10/10)	Male	CUMS	NM	2.22	42	SPT; FST; OFT
Yang et al. (2022) [33]	C57BL/6J (12/12)	Male	CUMS	pentobarbital	0.658	21	BW; FI; SPT; FST; OFT
Xiong et al. (2022) [34]	SD (6/6)	Male	CUMS	NM	38.54; 19.27; 9.635	21	BW; SPT; OFT
Lan et al. (2022) [35]	SD (10/10)	Male	CUMS	10% Chloral hydrate	16.70; 8.35; 4.175	42	BW; FI; OFT
Zhou et al. (2022) [36]	SD (25/25)	Male	CUMS	NM	2.224	21	BW; FI; SPT; OFT

Abbreviations: CUMS: Chronic Unpredictable Mild Stress; NM, not mentioned; BW, Body Weight; FI, Food Intake; SPT, Sucrose Preference Test; FST, Forced Swimming Test; NSF, Novelty Suppressed Feeding; OFT, Open Field Test.

**Table 2:** Convergence of mechanistic targets bridging meta-analysis and mechanism exploration driven by the same underlying immunomodulatory network.

Mechanism Target	Meta-Analysis Findings	Experimental Findings
Neuroinflammation	Reduces pro-inflammatory factors (IL-6, TNF-α) in the hippocampus and serum.	Targets the NF-κB pathway to inhibit TNF-α and IL-1β secretion.
Glial Cells Responses	Regulates astrocyte function and upregulates glial fibrillary acidic protein (GFAP).	Suppresses hyperactivation of microglia (IBA-1) and astrocytes (GFAP).
Systemic Regulation	Regulates the HPA axis (lowering ACTH/CORT) and suppresses glutamate.	Protects dopaminergic neurons, upregulates DA, and inhibits pituitary PRL secretion.

## Data Availability

The raw data supporting the conclusions of this article will be made available by the authors on request.

## Supplementary Materials

The following supporting information can be downloaded at: https://www.mdpi.com/article/10.3390/biomedicines14102270/s1.
