# Supplementary material for: Modified Xiaoyaosan Regulates the Neuro-Immune Microenvironment in Chronic Stress: A Preclinical Meta-Analysis and NF-kappaB Pathway Mechanism Exploration
Source: Biomedicines. 2026 Oct 8;14(10):2270. doi: 10.3390/biomedicines14102270 (PMC13701343; doi:10.3390/biomedicines14102270)

Supplementary Materials

Supplementary Methods

Phase 1: Meta-Analysis

1. Search Strategy

We preliminarily screened the relevant studies that may be related to the treatment of depression with Xiaoyaosan in Web of Science, PubMed, Cochrane Library, EMbase, VIP medicine information system (VMIS), China National Knowledge Infrastructure (CNKI), Chinese Biological Medical (CBM) and Wan-Fang. The literature retrieval time is up to June 2026, and the language is limited to English and Chinese. Search entries include: ("Xiaoyaosan" [MESH] OR "Xiaoyao Pill" [MESH] OR "Modified Xiaoyao San" [MESH] OR Xiaoyaosan Decoction [MESH] OR "Xiaoyao Decoction" [MESH] OR "Xiaoyao Formula" [MESH]) AND ("Depressed" [MESH] OR "Depression" [MESH] OR "Anti-depression" [MESH] OR "Chronic unpredictable mild stress" [MESH] OR "Unpredictable chronic mild stress" [MESH] OR "Antidepressant" [MESH] OR "Depressive-like behavior" [MESH] OR "Hyperprolactinemia" [MESH]).

2. Literature selection

The criteria for inclusion are as follows: (1) Population (P): Animal models of depression established by CUMS; (2) Intervention (I): Continuous treatment of Xiaoyaosan; (3) Control (C): the model group did not receive any treatment; (4) Outcome (O): The primary outcomes were body weight, food intake, sucrose preference test (SPT), open field test (OFT), forced swimming test (FST) and Novelty suppressed feeding (NSF), while the protective mechanism of Xiaoyaosan on depression was taken as the secondary outcome index.

The criteria for exclusion are as follows: (1) Population (P): not CUMS model; (2) Intervention (I): extracts from different parts of Xiaoyaosan or Xiaoyaosan combined with other drugs; (3) Control (C): the model group was treated with drugs; (4) Outcome (O): no one of the primary outcomes indicators or available data; (5) Not in vivo studies: review, meta-analysis, meeting abstract, clinical studies, in vitro studies; (6) Repeatedly published literature; (7) Languages other than English and Chinese.

3. Data extraction

The data of the inclusion study was extracted independently by two researchers, including (1) the first author's name and publication year; (2) the species, numbers and sex of animals; (3) the type of anesthetics in the experiment; (4) modeling time and administration time, intervention dose, administration cycle and administration route; (5) outcome index.

The maximum dose group data is extracted from each outcome index. When the result is obtained through multiple time points, we only extract the data from the last time point. For the graphic results, the mean and standard deviation (SD) are obtained by WebPlotDigiizer correlation measurement.

4. Methodological quality evaluation

The included study was independently evaluated by two researchers according to the Collaborative Approach to Meta-Analysis and Review of Animal Data from Experimental Studies (CAMARADES) 10-point scoring scale. In order to adapt to our research, the quality check list has been improved (G: avoidance of anesthetic with significant intrinsic neuroprotective activity or neurotoxicity). When there is a disagreement, discuss with another author.

5. Statistical analysis

The outcome index was analyzed by Stata software (version 13.0, Stata Corporation, College Station, TX, USA). Results the average difference (MD) was calculated with 95% confidence interval (CI), and the heterogeneity of the data was evaluated by I^2 test. If $I^2<50\%$ or $p>0.05$, it is considered that there is no heterogeneity, and the fixed effect model is used to analyze. Otherwise, the heterogeneity is considered to exist and the random effect model is adopted. Sensitivity analysis was used to investigate the effects of various studies on the overall Meta analysis. Funnel chart and Egger's test were used to analyze the publication bias of the selected study.

All quantitative data are presented as the mean ± standard deviation (SD). To ensure adequate statistical power for our in vivo and molecular endpoints, an a priori power analysis was conducted using STATA version 13.0. Based on comparable preclinical neuro-immune evaluations, a sample size of n = 6 for molecular assays (Western blot, PCR, IHC) and n = 20 for behavioral/physiological tracking was determined sufficient to detect a large effect size (Cohen's f = 0.40) with an α error probability of 0.05 and a statistical power (1 - β) of 0.80.

Prior to hypothesis testing, the normality of the data distribution for all variables was assessed using the

Shapiro-Wilk test, confirming that the data met the assumptions for parametric testing. Outlier detection was formally conducted using the ROUT method (Q = 1%); no data points were identified as significant outliers, and therefore no data were excluded from the final analyses.

For comparisons involving multiple independent groups at a single time point, the rationale dictated the use of a One-Way Analysis of Variance (ANOVA). When the ANOVA indicated a significant omnibus effect, Tukey's post-hoc test was applied to control the family-wise error rate during multiple pairwise comparisons. For continuous variables measured repeatedly over time (e.g., body weight tracking), a Repeated Measures ANOVA was utilized to account for intra-subject correlation. All statistical analyses and graphical visualizations—which display individual data points for transparency—were generated using GraphPad Prism version 9.0. A p-value < 0.05 was considered statistically significant.

Supplementary Results

Phase 1 Meta-Analysis: Xiaoyaosan Improves Behavioral Despair and Anhedonia in CUMS Models

1. Research screening

According to the retrieval strategy, a total of 3019 pieces of data were retrieved. After excluding repetitive and irrelevant literatures, there were 124 articles left. After further excluding the literatures of reviews, meeting reports, scientific and technological achievements, abstracts (n = 4), network pharmacology (n = 3) and the effective parts of Xiaoyaosan (n = 14), 103 studies were included in further screening. Non-CUMS models (n = 44), non-pharmacodynamic experimental studies (n = 15) and in vitro studies (n = 1) were excluded by reading titles and abstracts. 43 articles were read in full, and those with duplicate data of journals and master's degree papers (n = 10) and those that met the inclusion criteria but the data were not available (n = 8) were excluded, and 25 studies were included for comprehensive analysis, including Dai et al. (2010)[14], Jia et al. (2011)[15], Tan et al. (2015)[16], Chen et al. (2015)[17], Ding et al. (2017)[18], Guo et al. (2017)[19], Yan et al. (2018)[13], Liu et al. (2019)[20], Xu et al. (2019)[21], Lv et al. (2020)[22], Liu et al. (2020)[23], Wang et al. (2020)[24], Wan et al. (2020)[25], Ding et al. (2020)[26], Song et al. (2020)[12], Yan et al. (2021)[27], Lv et al. (2021)[28], Zhou et al. (2021)[29], Liang et al. (2021)[30], Jiao et al. (2021)[31], Yuan et al. (2022)[32], Yang et al. (2022)[33], Xiong et al. (2022)[34], Lan et al. (2022)[35], and Zhou et al. (2022)[36] **(Fig.**

2).

Research characteristics

The basic characteristics of the 25 literatures are shown in the **Table 1**. A total of 569 animals were included in this study, of which 68% were Sprague-Dawley (357/569), 4% were Wistar (24/569), and 28% were C57BL/6J (188/569) **(Fig. S1.A)**. All rodents were male **(Fig. S1.B)**. The sample size for each study ranges from 6 to 25. Pentobarbital sodium (28%, 7/25), chloral hydrate (12%, 3/25) and decapitation (4%, 1/2) were mainly used as anesthetics, and anesthetic use was not reported in 14 studies **(Fig. S1.C)**.

All studies were modeled with chronic unpredictable stimuli and treated with Xiaoyao san, with a dose range of 0–92.4 g/kg, divided into three dose groups (0–10 g/kg: low dose, 10–20 g/kg: medium dose, 20–100 g/kg: high dose), low dose 72%, medium dose 20%, high dose 24%. 5 studies included multiple dose groups. The administration methods were via gavage orally (100%, 25/25) (Fig. 1.2D), and the administration cycle involved 21d (52%, 13/25),28d (28%, 7/25),42d (16%, 4/25),49d (4%, 1/25) **(Fig. S1.E)**.

With outcome indicators, 21 (84%) studies measured body weight, 11 (44%) reported changes in food intake, 24 (96%) reported sucrose preference test, and 4 (16%) reported novel feeding experiments. of these, 4 (16%) reported the results of delayed feeding and 2 (8%) reported food consumption. Forced swimming experiments were carried out in 10 (40%) studies. In the open field experiment, there were 6 (24%) reported crossing, 8 reported rearing, 7 (28%) reported the number of entries into the center zone, 14 (56%) reported total running distance, 11 (44%) reported time spent in center area, 3 (12%) reported the number of clean.

Quality evaluation

The modified Camarades10-item checklist was used to evaluate the methodological quality of the 25 studies. The score ranged from 5/10 to 9/10, with an average score of 7.48.

Specifically, all studies used appropriate CUMS animal models, calculated the sample size, and were peer-reviewed. Three studies did not indicate whether to use constant temperature control, including Jia et al., 2011[15], Tan et al., 2015[16], and Chen et al., 2015[17]. All studies mentioned random allocation and distribution hiding, but none of the studies blindly evaluated the results. 11 studies

avoided the use of neuroprotective or toxic anesthetics. 64% of the studies showed compliance with animal welfare principles, and 52% of the studies stated that there was no conflict of interest. The specific risks of bias items are shown in **Fig.S1.F** and **Fig.S2**.

Body weight and food intake

A total of 21 studies reported body weight as an outcome indicator. The results showed that the body weight of CUMS group decreased, and after Xiaoyao san treatment was significantly higher than that of CUMS group. (SMD=2.65, 95%CI (2.13, 3.17), p = 0.000, heterogeneity: I2 =75.1%) **(Fig. S3.A)**.

11 studies reported food intake levels. As shown in **Fig. S3.B**, there is significant heterogeneity between the two groups of studies (p = 0.000, I2 = 87.6%), using a random effect model. The results showed that Xiaoyaosan could significantly increase animal appetite and restore food intake (SMD = 4.88, 95%CI (3.62, 6.13), p = 0.000). Sensitivity analysis showed that the combined effect points of all studies fell within the confidence interval of the total effect, suggesting that the results of body weight and food intake were reliable **(Fig. S4)**.

SPT

SPT is considered to be the most common way to observe the loss of pleasure caused by depression. SPT was reported in 24 studies. The results of heterogeneity showed that there was significant heterogeneity in the study (p = 0.000, I^2 = 89.9%), which was analyzed by random effect model. Compared with CUMS group, Xiaoyaosan could significantly increase the preference for sucrose (SMD = 3.43, 95%CI (2.62, 4.23), p = 0.000) **(Fig. S5.A)**. Sensitivity analysis showed that the results were robust **(Fig. S5.B)**.

FST and NSF

The results of meta-analysis of 9 studies showed that Xiaoyaosan could significantly reduce the immobility time in forced swimming compared with CUMS group (SMD = -4.97, 95%CI (-6.29, -3.65), p=0.000, heterogeneity: I2 = 82.0%) **(Fig. S6.A)**. Sensitivity analysis showed that the results of FST were reliable **(Fig. S7.A)**.

4 articles reported delayed feeding in NSF. Meta analysis showed that Xiaoyaosan could significantly shorten the latent period of eating (SMD = -2.12, 95%CI (-3.15, -1.08), p = 0.000, heterogeneity: I2

=72.7%) **(Fig. S6.B)**. The meta results of 2 studies showed that there was no significant difference in food consumption after inhibition between the two groups (SMD =-2.74, 95%CI (-0.59, 6.07), p = 0.107, heterogeneity: I2 = 93.1%) **(Fig. S6.C)**. Sensitivity analysis shows that NFS's conclusion is robust **(Fig. S7.B)**.

OFT

14 studies reported total exercise distance. The results showed that the total running distance of Xiaoyaosan group was significantly higher than that of CUMS group (SMD = 3.96, 95%CI (2.90, 5.02), p = 0.000, heterogeneity: I2 = 89.4%) **(Fig. S8.A)**. 7 articles analyzed the number of entries into the central zone, and the results of meta-analysis showed that the number of times of crossing the center after treatment of Xiaoyaosan was significantly higher than that of CUMS group (SMD =2.78, 95%CI (1.06, 4.50), p = 0.000, heterogeneity: I2 = 93.5%) **(Fig. S8.B)**. The meta results of 6 studies showed that Xiaoyaosan could significantly increase the number of crossing lattices compared with CUMS group (SMD = 1.6, 95%CI (0.77, 2.43), p = 0.000, heterogeneity: I2 = 63.2%) **(Fig. S8.C)**.

8 studies reported the number of upright times. There is no heterogeneity among the studies, so the fixed effect model is used (p = 0.115, I2 = 39.5%). The results showed that the number of upright in Xiaoyaosan group was significantly higher than that in CUMS group (SMD =1.34, 95%CI (0.93, 1.75), p = 0.000) **(Fig. S9.A)**. The results of meta-analysis of 3 studies showed that there was no significant difference in cleaning times between Xiaoyaosan and CUMS group (SMD = 1.62, 95%CI (− 0.17, 3.41), p = 0.076, heterogeneity: I2 = 89.2%) **(Fig. S9.B)**. The sensitivity analysis results of the total running distance, the times of entering the central area, the number of crossing lattices, the times of upright and the times of cleaning show that the results are reliable **(Fig. S10)**.

Heterogeneity and results of subgroup analysis

Two main indexes of body weight and SPT were selected for egger analysis. The results of Egger analysis showed that there was a certain publication bias in the study (body weight: $p>|t| = 0.002 < 0.05$; SPT: $p > |t| = 0.00 < 0.05$) **(Fig. S11)**. Next, the main sources of heterogeneity are further analyzed.

Some potential factors, such as different dose, different animal species and different administration time, may increase the heterogeneity of the results. In this meta-analysis, subgroup analysis was used to explore the potential evidence of heterogeneity sources.

For body weight, based on the subgroup analysis of different doses of Xiaoyao san, it was found that the heterogeneity of middle and high dose groups decreased significantly, while the heterogeneity of low dose group was still at a high level **(Fig. S12.A)**, indicating that the dose of Xiaoyaosan was the main source of heterogeneity. For different species, the heterogeneity of rat group decreased slightly, but there was no significant difference between rat group and mouse group **(Fig.S12.B)**. The heterogeneity analysis caused by different administration time showed that the heterogeneity decreased significantly in 28 days group, but there was no significant change in 21 days and 42 days groups. 49 days after administration, only one study was not examined **(Fig.S12.C)**.

Similarly, the heterogeneity sources of SPT results were analyzed. In the analysis of different doses, it was found that the heterogeneity of the results was significantly reduced in the middle dose group, the heterogeneity in the high dose group decreased to a certain extent, and the heterogeneity in the low dose group remained at a high level **(Fig.S13.A)**, suggesting that the dose of Xiaoyaosan was the main cause of heterogeneity. Secondly, different species had no significant effect on the heterogeneity of the results **(Fig.S13.B)**. In the subgroup analysis of different administration time, it was found that 28 days and 42 days could significantly reduce the heterogeneity of the results, but there was no significant change in the 21 days group **(Fig.S13.C)**.

Supplementary Figure Legends

Figure.S1 Included study characteristics.

Note: (A) model species; (B) sex; (C) anesthetic; (D) method of administration, (E) duration of intervention, (F) research quality score.

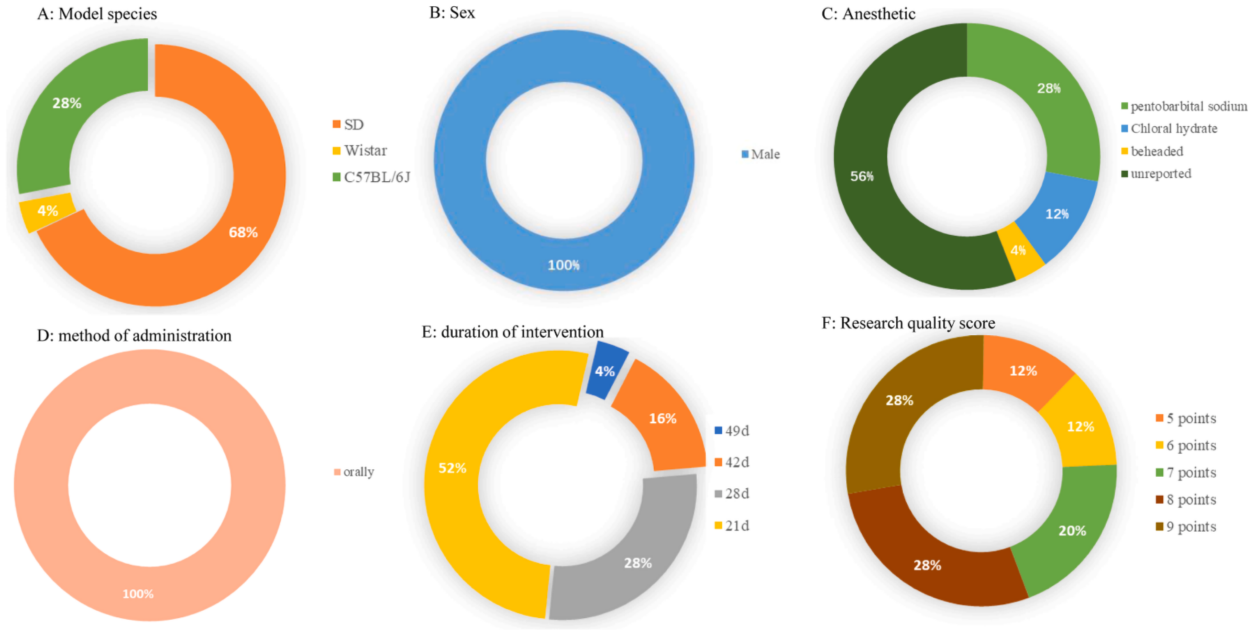

Figure.S2 Risk of bias and quality score of included studies.

Note: A, appropriate animal model; B, same size calculation; C, control of temperature; D, random allocation to treatment or model; E, allocation concealment; F, blinded assessment of outcome; G, avoidance of anesthetic with significant intrinsic neuroprotective activity or neurotoxicity; H, compliance with animal welfare regulation; I, statement of potential conflict of interests; J, peer reviewed published.

first author	publication year	A	B	C	D	E	F	G	H	I	J	Total
Dai	2010	√	√	√	√	√			√		√	7
Jia	2011	√	√		√	√					√	5
Tan	2015	√	√		√	√					√	5
Chen	2015	√	√		√	√					√	5
Ding	2017	√	√	√	√	√		√	√	√	√	9
Guo	2017	√	√	√	√	√		√		√	√	8
Yan	2018	√	√	√	√	√		√	√	√	√	9
Liu	2019	√	√	√	√	√		√	√	√	√	9
Xu	2019	√	√	√	√	√			√		√	7
LV	2020	√	√	√	√	√					√	6
Liu	2020	√	√	√	√	√					√	6
Wang	2020	√	√	√	√	√					√	6
Wan	2020	√	√	√	√	√		√			√	7
Ding	2020	√	√	√	√	√			√	√	√	8
Song	2020	√	√	√	√	√			√	√	√	8
Yan	2021	√	√	√	√	√		√	√	√	√	9
LV	2021	√	√	√	√	√			√	√	√	8
Zhou	2021	√	√	√	√	√		√	√	√	√	9
Liang	2021	√	√	√	√	√		√	√		√	8
Jiao	2021	√	√	√	√	√		√	√	√	√	9
Yuan	2022	√	√	√	√	√			√	√	√	8
Yang	2022	√	√	√	√	√		√	√	√	√	9
Xiong	2022	√	√	√	√	√			√	√	√	8
Lan	2022	√	√	√	√	√		√			√	7
Zhou	2022	√	√	√	√	√			√		√	7

Figure.S3 Effects of Xiaoyao san on body weight (A) and food intake (B).

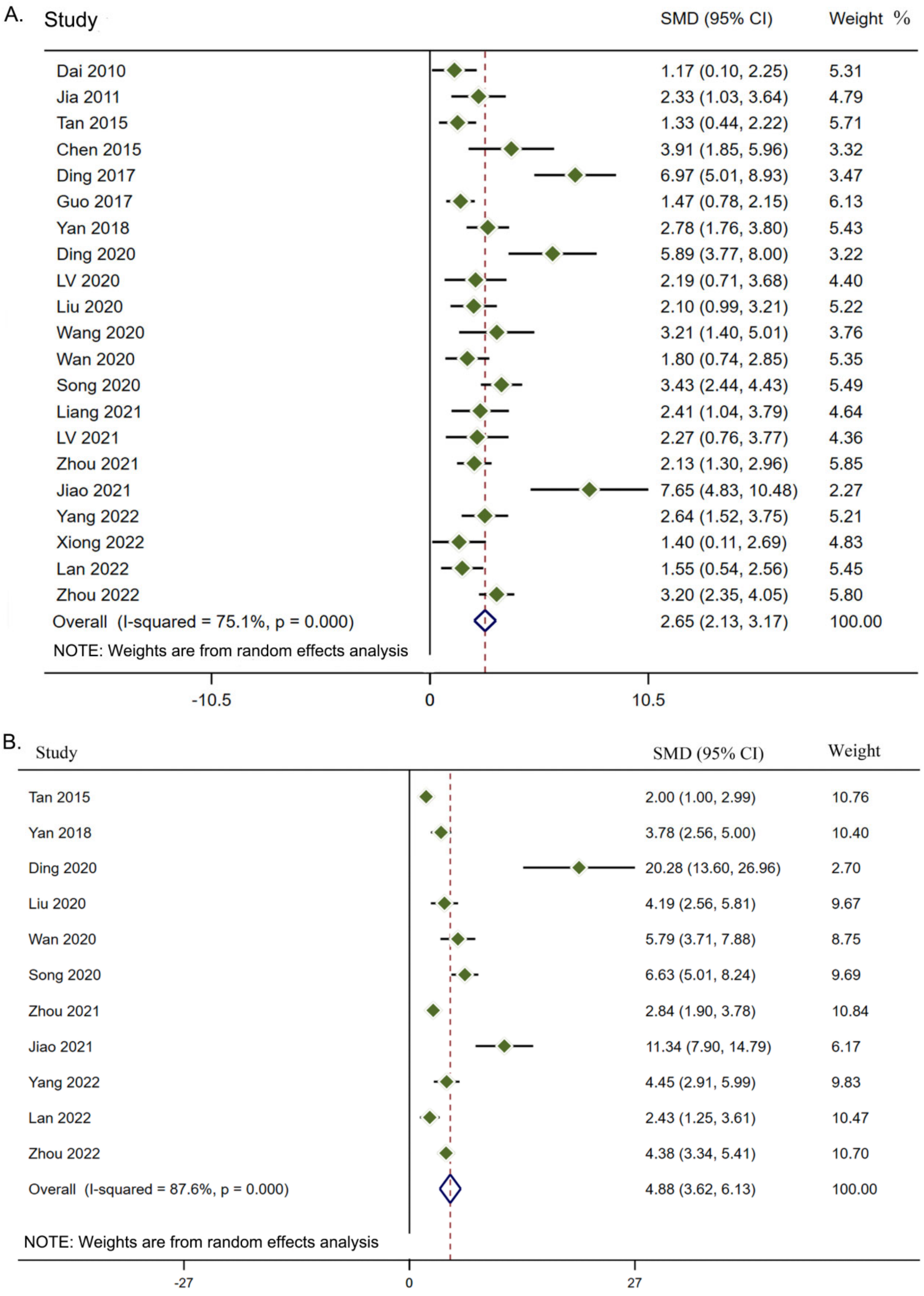

Figure.S4 Sensitivity analysis of body weight (A) and food intake (B).

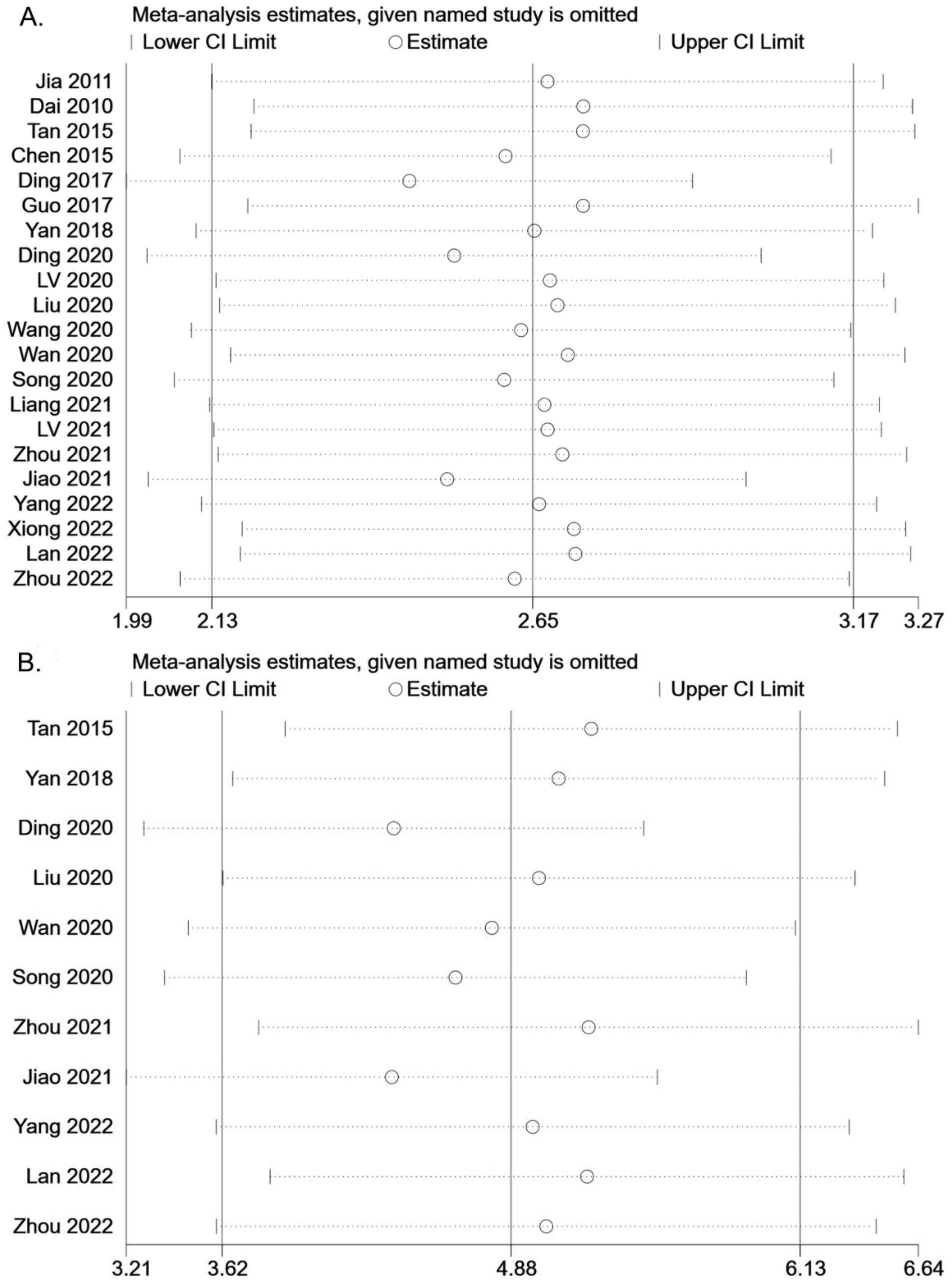

Figure.S5 Forest map (A) and sensitivity analysis (B) of SPT.

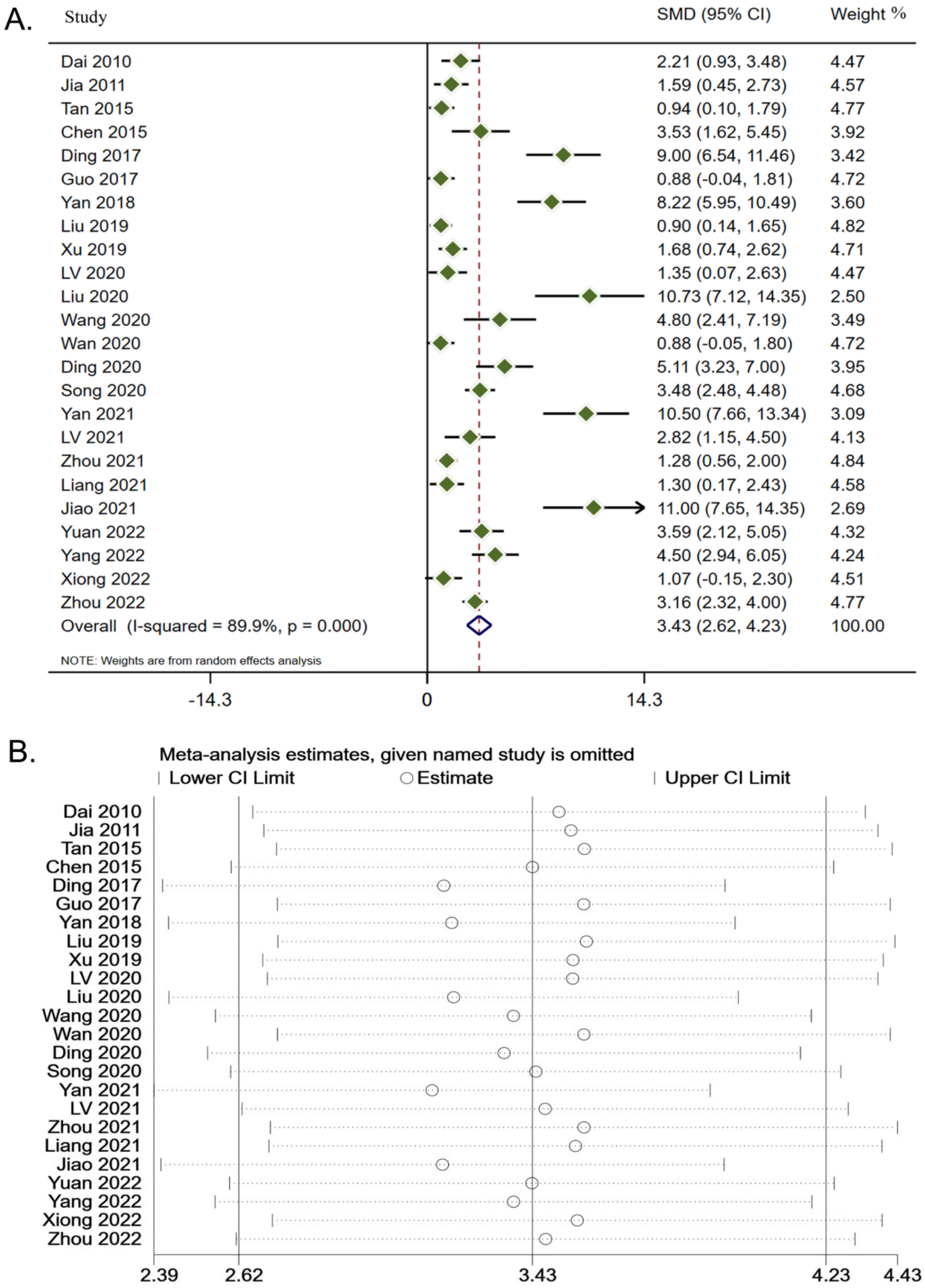

Figure.S6 Influence of Xiaoyao san on FST (A) and NST (B: latency to feed, C: food consumption).

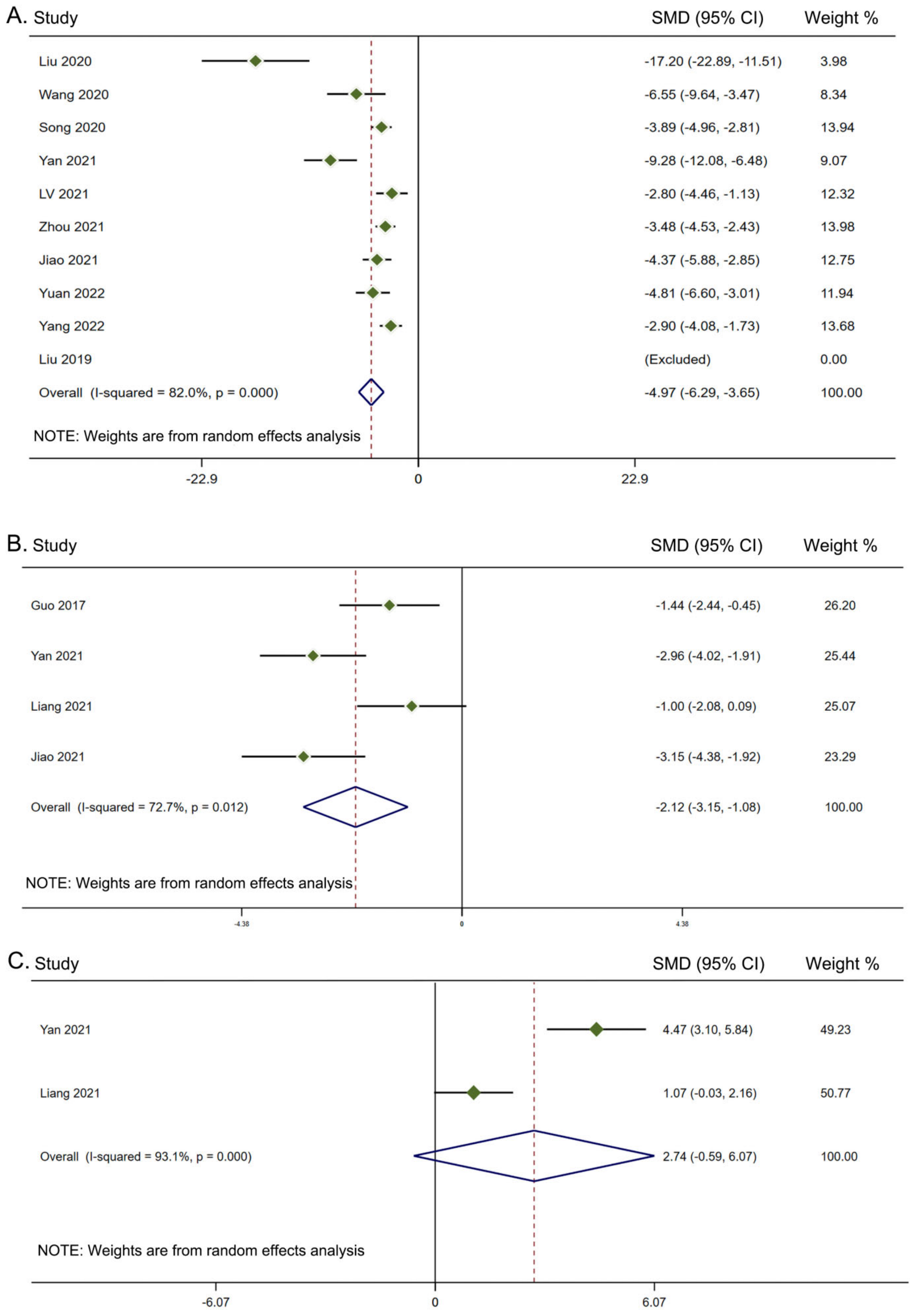

Figure.S7 Sensitivity analysis of FST and NST.

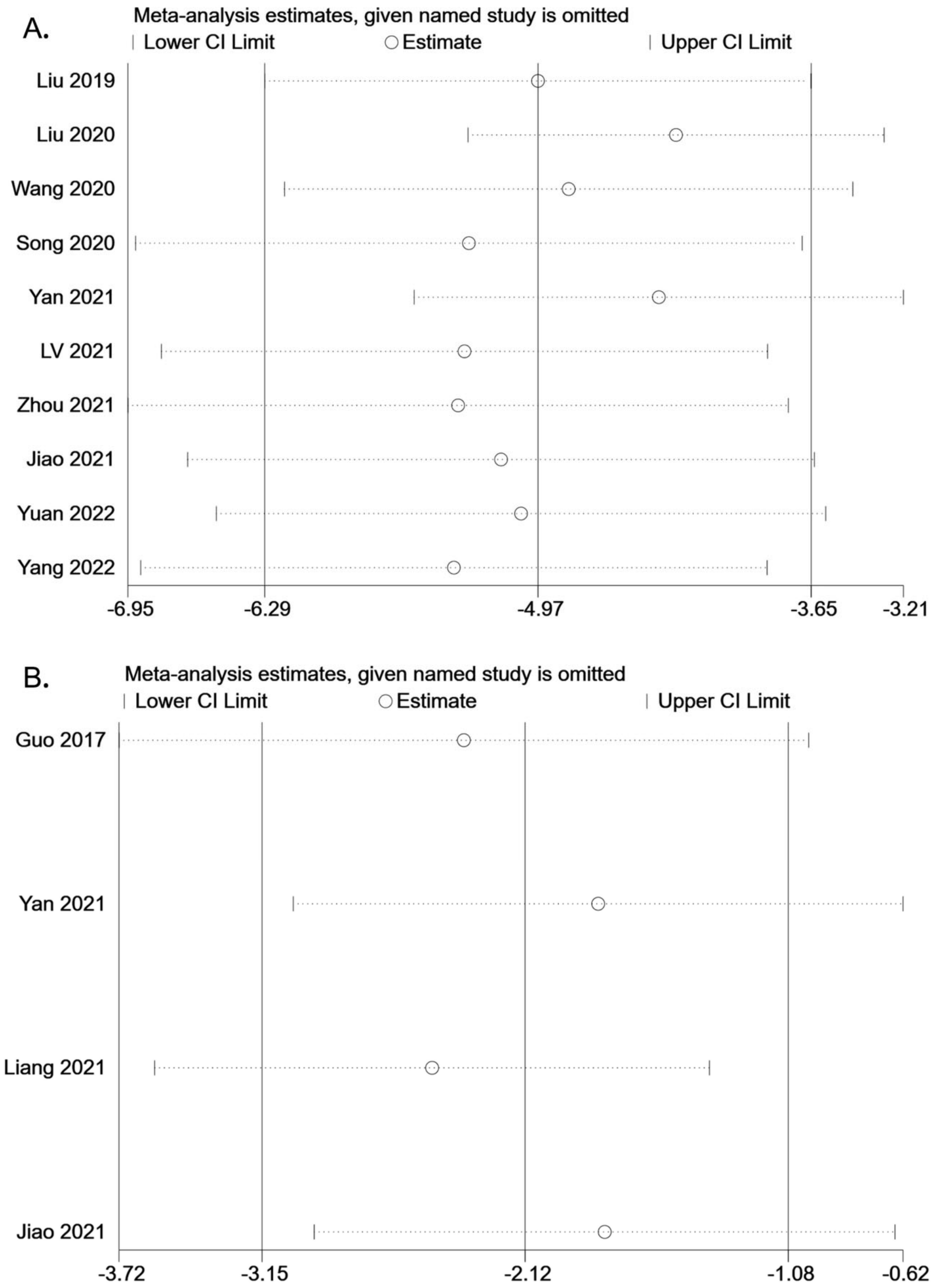

Figure.S8 The influence of Xiaoyao san on the total running distance (A), the number of entries into the central zone (B) and the number of crossing lattices (C) in OFT.

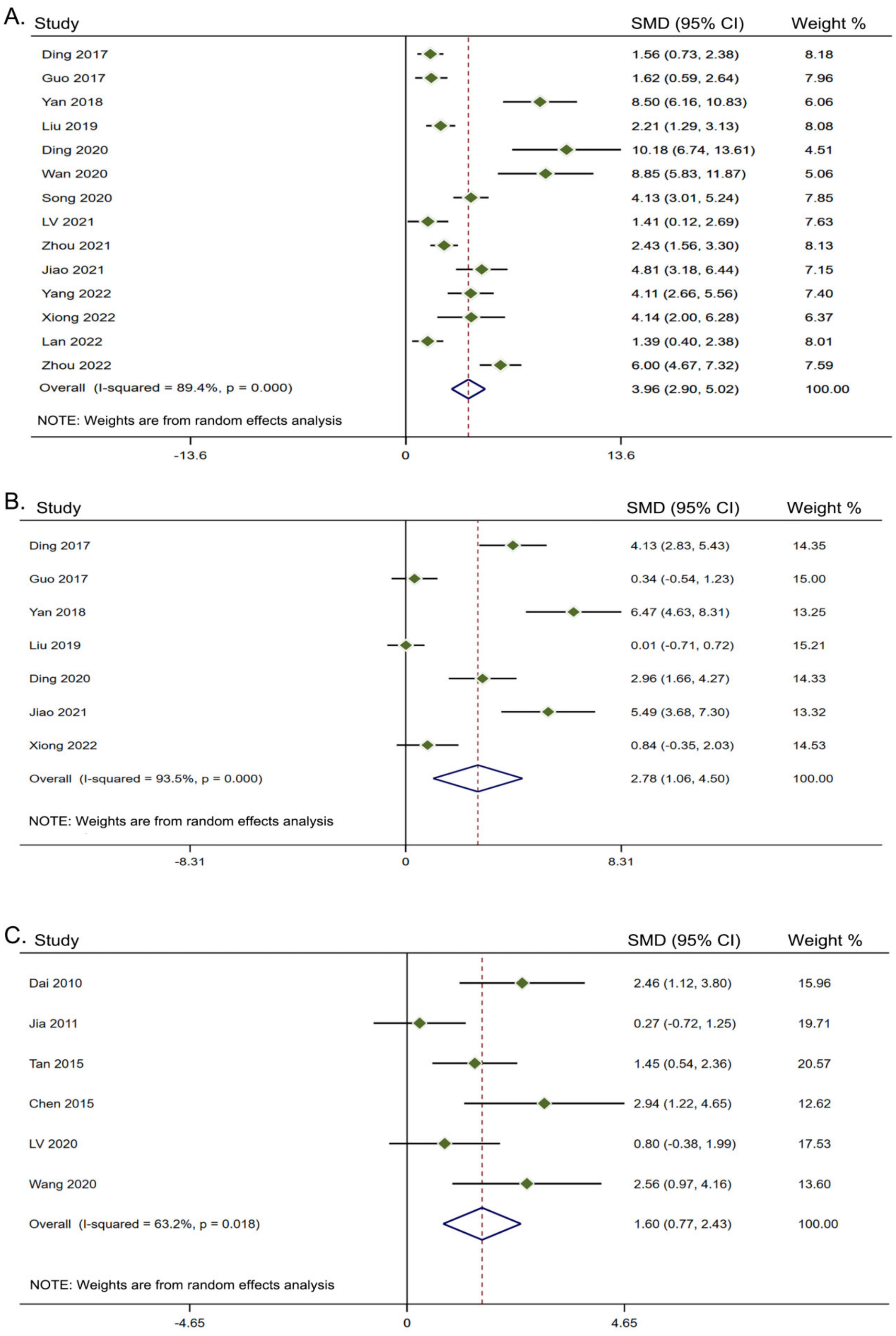

Figure.S9 The influence of Xiaoyao san on the number of upright times (A), the number of cleaning times (B).

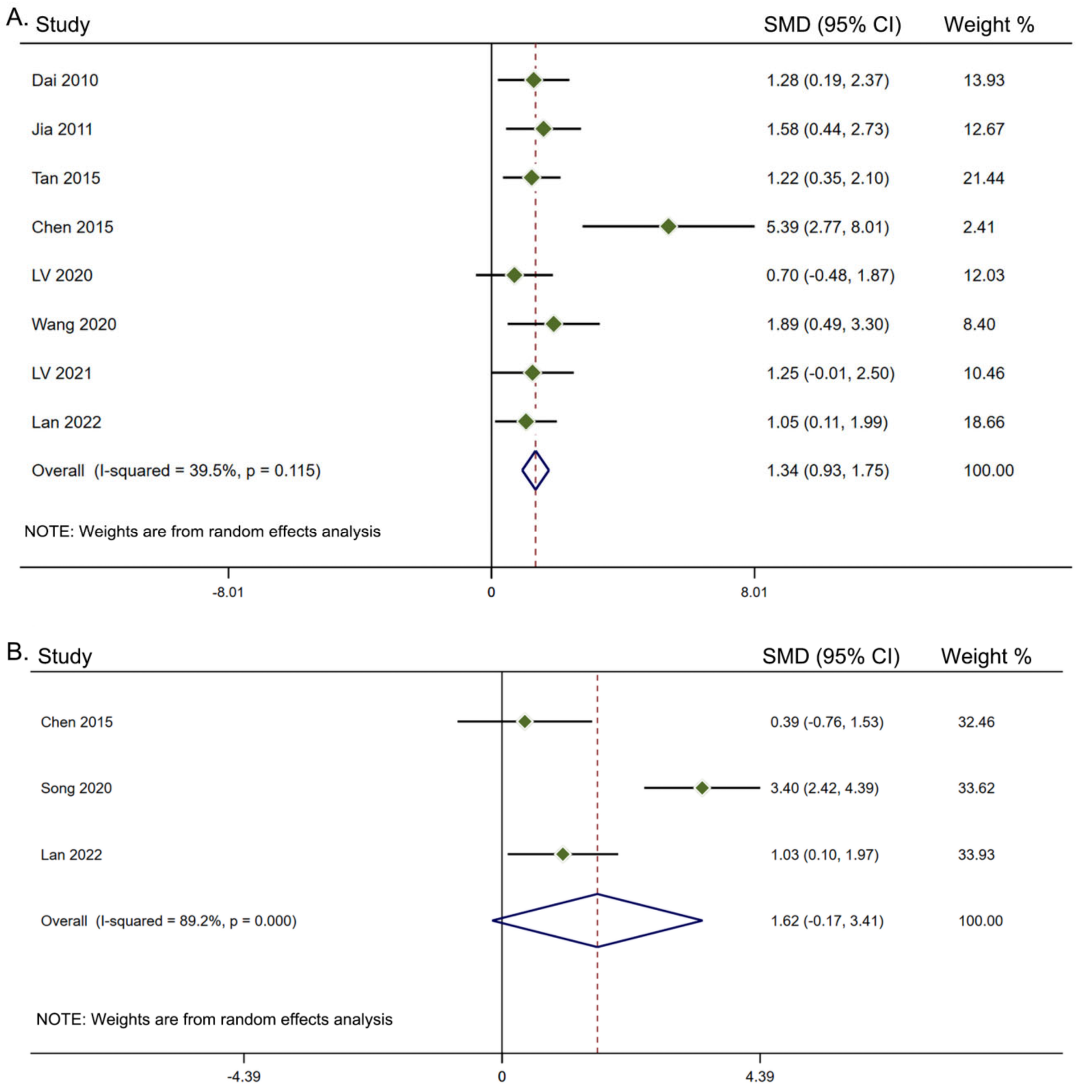

Figure.S10 Sensitivity analysis of OFT results. (A) the total running distance, (B) the number of entries into the central zone, (C) the number of crossing lattices, (D) the number of upright times and (E) the number of cleaning times.

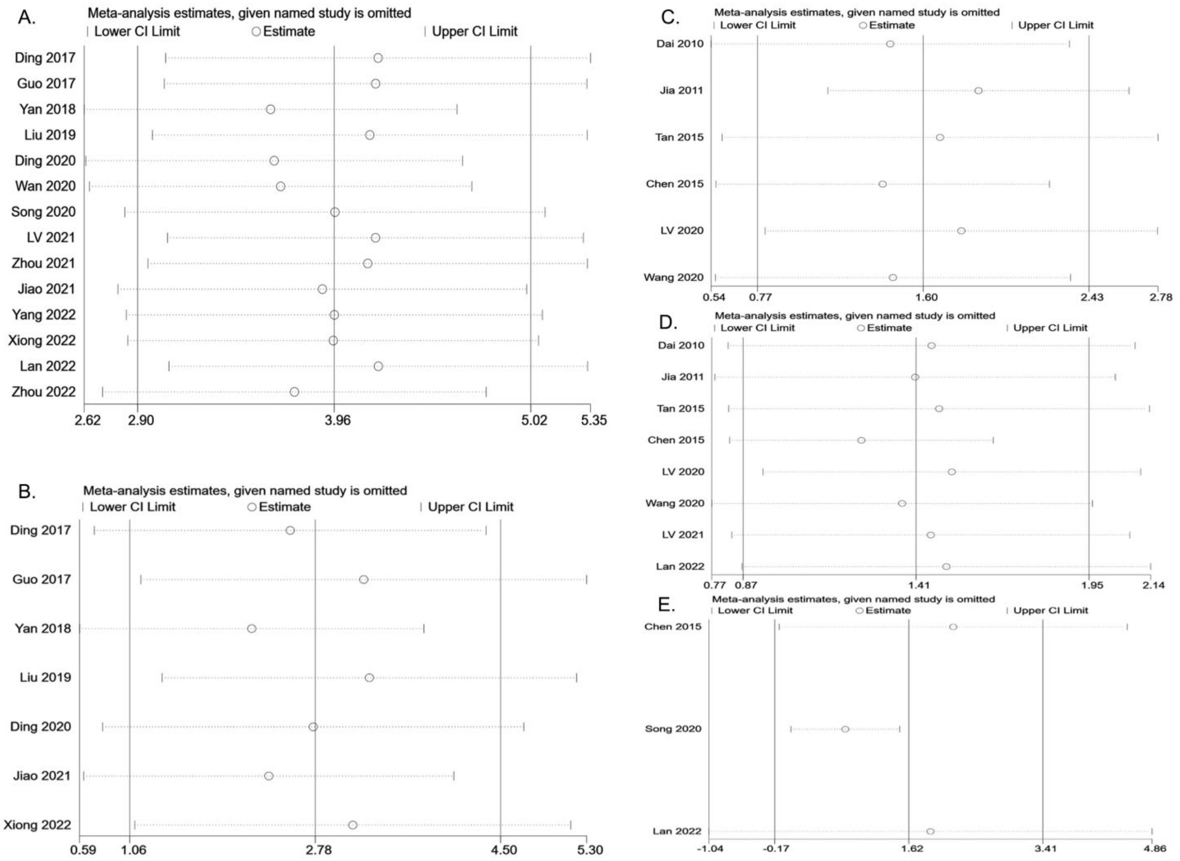

Figure.S11 Egger analysis of body weight (A) and SPT (B).

A.

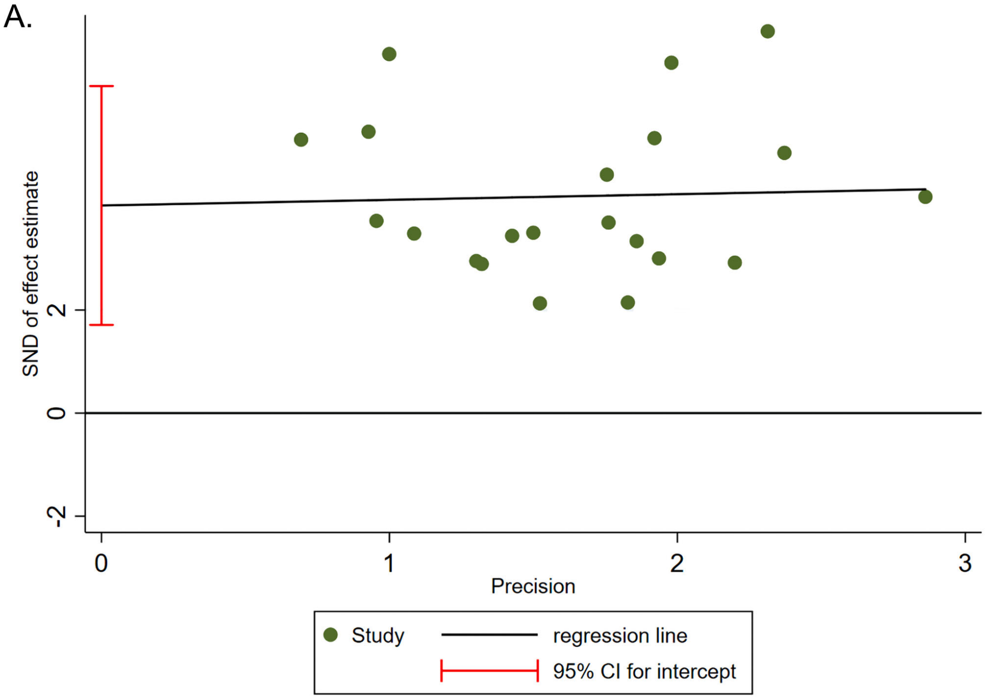

B.

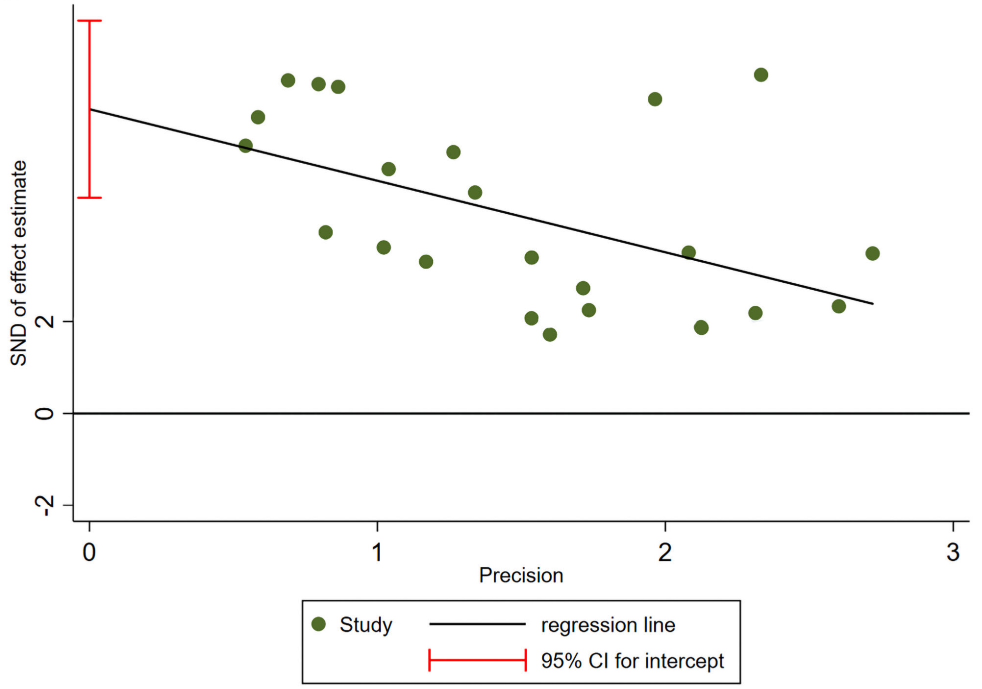

Figure.S12 Stratified analysis of body weight according to dosage of Xiaoyao san (A), animal species (B), and administration time (C).

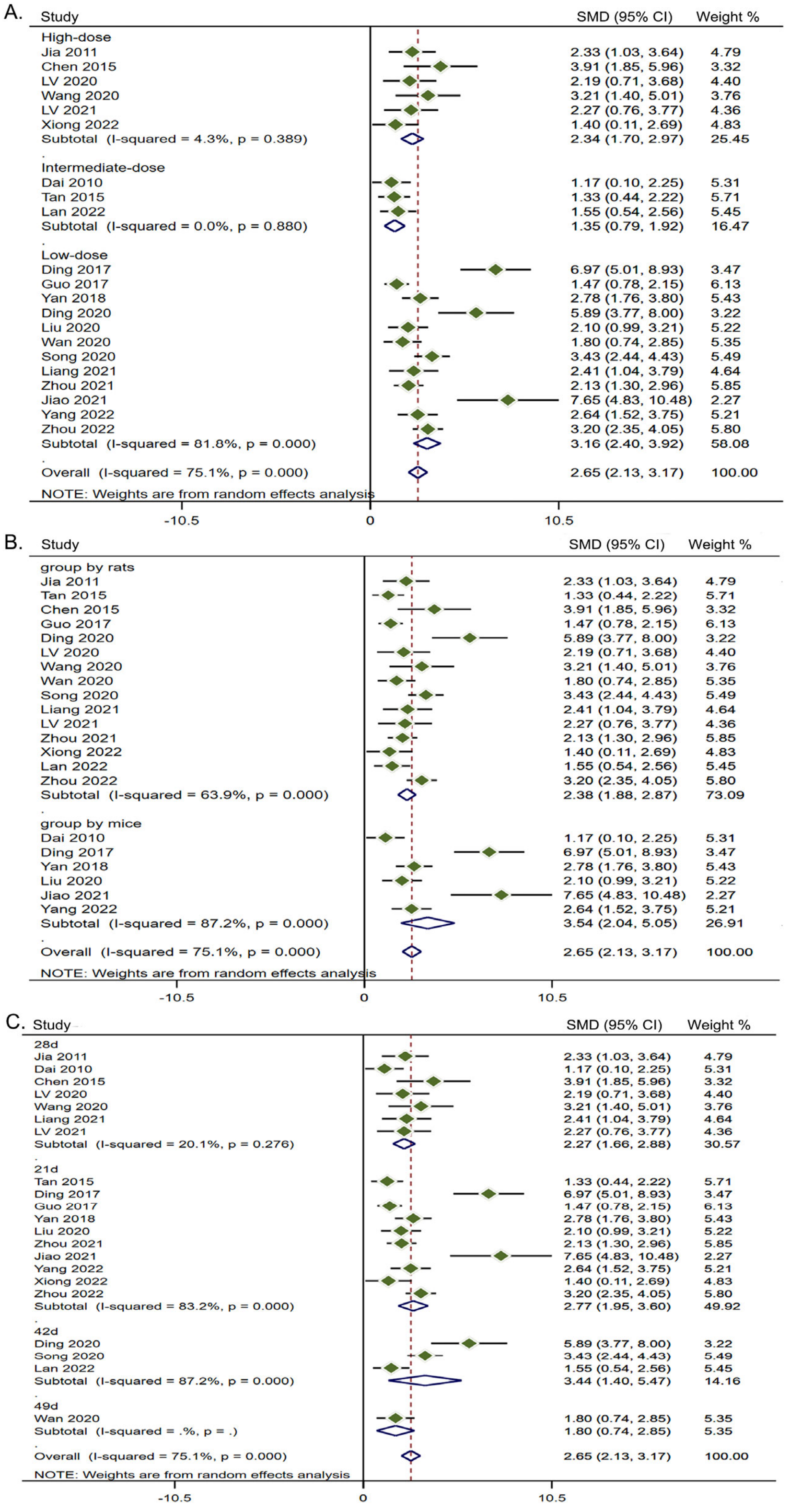

Figure.S13 Stratified analysis of SPT according to dosage of Xiaoyao san (A), animal species (B), and administration time (C).

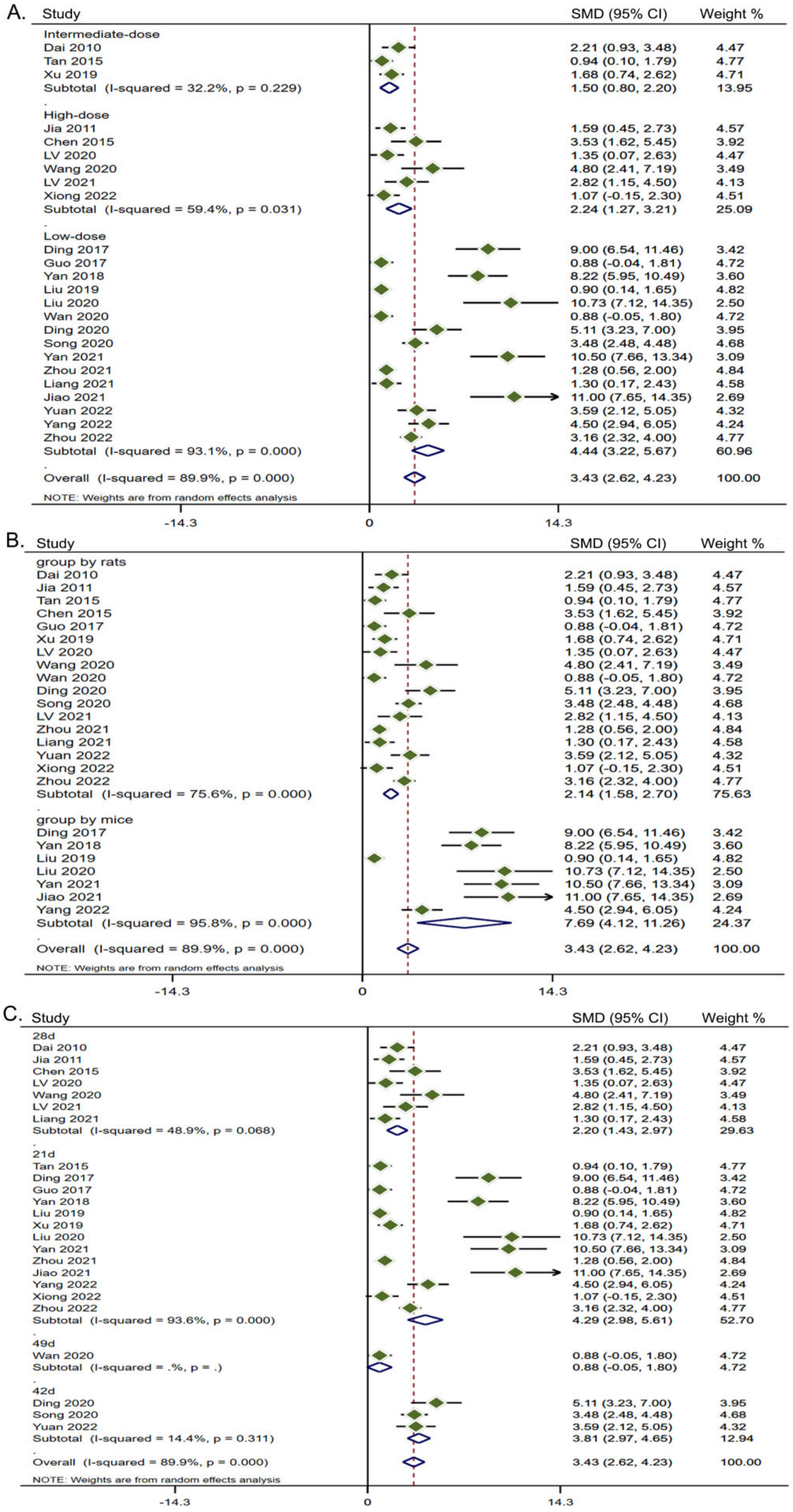

Supplement: Supplementary file 1 [file biomedicines-14-02270-s001.zip › biomedicines-4559143-supplementary.pdf]
